# Cholangiocarcinoma: Correlation between Molecular Profiling and Imaging Phenotypes

**DOI:** 10.1371/journal.pone.0132953

**Published:** 2015-07-24

**Authors:** Eran Sadot, Amber L. Simpson, Richard K. G. Do, Mithat Gonen, Jinru Shia, Peter J. Allen, Michael I. D’Angelica, Ronald P. DeMatteo, T. Peter Kingham, William R. Jarnagin

**Affiliations:** 1 Department of Surgery, Memorial Sloan Kettering Cancer Center, New York, New York, United States of America; 2 Department of Radiology, Memorial Sloan Kettering Cancer Center, New York, New York, United States of America; 3 Department of Epidemiology and Biostatistics, Memorial Sloan Kettering Cancer Center, New York, New York, United States of America; 4 Department of Pathology, Memorial Sloan Kettering Cancer Center, New York, New York, United States of America; National Cancer Institute, UNITED STATES

## Abstract

**Purpose:**

To investigate associations between imaging features of cholangiocarcinoma by visual assessment and texture analysis, which quantifies heterogeneity in tumor enhancement patterns, with molecular profiles based on hypoxia markers.

**Methods:**

The institutional review board approved this HIPAA-compliant retrospective study of CT images of intrahepatic cholangiocarcinoma, obtained before surgery. Immunostaining for hypoxia markers (EGFR, VEGF, CD24, P53, MDM2, MRP-1, HIF-1α, CA-IX, and GLUT1) was performed on pre-treatment liver biopsies. Quantitative imaging phenotypes were determined by texture analysis with gray level co-occurrence matrixes. The correlations between quantitative imaging phenotypes, qualitative imaging features (measured by radiographic inspection alone), and expression levels of the hypoxia markers from the 25 tumors were assessed.

**Results:**

Twenty-five patients were included with a median age of 62 years (range: 54–84). The median tumor size was 10.2 cm (range: 4–14), 10 (40%) were single tumors, and 90% were moderately differentiated. Positive immunostaining was recorded for VEGF in 67% of the cases, EGFR in 75%, and CD24 in 55%. On multiple linear regression analysis, quantitative imaging phenotypes correlated significantly with EGFR and VEGF expression levels (R^2^ = 0.4, *p*<0.05 and R^2^ = 0.2, *p*<0.05, respectively), while a trend was demonstrated with CD24 expression (R^2^ = 0.33, *p* = 0.1). Three qualitative imaging features correlated with VEGF and CD24 expression (P<0.05), however, none of the qualitative features correlated with the quantitative imaging phenotypes.

**Conclusion:**

Quantitative imaging phenotypes, as defined by texture analysis, correlated with expression of specific markers of hypoxia, regardless of conventional imaging features.

## Introduction

Radiogenomics is an emerging field focusing on establishing relationships between imaging phenotypes and molecular markers utilizing novel methods [1,2]. Advances in radiogenomic imaging have the potential to contribute to clinical decision making through development of predictive and prognostic treatment algorithms and noninvasive disease surveillance. This promising approach has several advantages compared to the current molecular profiling methods. The latter require invasive tissue procurement procedures that lack temporal and spatial dimensions, as they provide information in a single time point, typically from a single anatomical site. By contrast, the radiogenomic approach can be implemented in multiple time points and at multiple tumor sites.

As imaging technology continues to evolve, the ability to correlate imaging phenotype with tumor genotype will continue to improve, and the strength and clinical utility of these relationships will be enhanced. Texture analysis is a novel technique that measures heterogeneity of tumors by quantifying the spatial pattern of pixel intensities on cross sectional imaging [3]. Recent reports have demonstrated promising diagnostic and prognostic performance of texture analysis in colorectal cancer [4], brain tumors [5], hepatic tumors [6], and hepatic dysfunction [7]. ICC, an aggressive primary liver cancer with a low but clearly documented increase in incidence and mortality [8], is characterized by frequent over-expression of epidermal growth factor receptor (EGFR), vascular endothelial growth factor (VEGF), as well as other pro-angiogenic and hypoxia mediators [9–14]. These molecular features of ICC result in marked distortion of the microvascular phenotype, which combined with their frequent large size, make it an ideal tumor type for experimental studies that correlate quantitative imaging parameters and molecular profiling.

We hypothesized that heterogeneous tumor enhancement on imaging reflects regions of abnormal vasculature and hypoperfusion due to the hypoxic microenvironment, which is characterized by overexpression of hypoxia markers [15]. To address this question, we utilized a model of intrahepatic cholangiocarcinoma (ICC) to investigate the relationship between imaging phenotypes and a clinically-oriented molecular profile based on hypoxia markers. The imaging phenotype was determined by texture analysis of contrast enhanced computed tomography (CT) data, which quantifies the heterogeneity in tumor enhancement pattern.

## Materials and Methods

### Patients

Patients signed an informed consent that covered review of medical records and studies for correlated research. The study was approved by the Institutional Review Board (IRB) of Memorial Sloan Kettering Cancer Center (MSKCC). From August 2003 through September 2009, two phase II clinical trials (NCT00587067 and NCT00410956) evaluating the role of regional chemotherapy in patients with initially unresectable primary liver cancer (either ICC or hepatocellular carcinoma) were conducted at MSKCC [16,17]; all patients signed IRB-approved consent forms for participation in these trials. These studies included 56 patients (44 with ICC and 12 with hepatocellular carcinoma). Additional details of these studies, including preoperative assessment and inclusion/exclusion criteria, have been previously reported. As part of these studies, hepatic artery infusion pumps were placed intraoperatively and biopsies were taken concurrently from the peripheral aspect of the dominant tumor and non-tumor liver. Biopsies were not performed during the course of treatment. All patients with ICC enrolled in one of these two clinical trials were included in the current study (n = 44). Patients treated as part of both studies were combined in the outcome analyses since there were no differences in selection criteria, demographics, tumor characteristics, response rates, or survival [18]. Clinicopathologic data, prospectively collected during the clinical trials, were analyzed. No overlap exists between the prior trials and the current study, as the prior trials focused on the therapeutic role of regional chemotherapy for unresectable primary liver cancer whereas the current study reports on the diagnostic performance of CT to detect the molecular profile of ICC.

### Hypoxia markers and immunohistochemistry

Molecular profiling of tumors was based on immunohistochemistry studies targeting the following established hypoxia markers (S1 Table): VEGF[15], EGFR[15], MRP-1[19], HIF-1α[15], CA-IX[20], CD24[21], GLUT1[22], P53[23], and MDM2[24]. The details of the primary antibodies have been previously reported [25–27]. All immunostains were evaluated without any knowledge of the clinical findings by a dedicated liver pathologist (J.S.). Stains were graded as a continuous variable according to the percentage of positive tumor cells. For HIF-1α and CA IX, positive staining required positive nuclear labeling in >5% of tumor cells, and for VEGF, EGFR, P53, MDM2, CD24, MRP-1, and GLUT1, positive staining refers to cytoplasmic and/or membranous labeling in >10% cells. These cut-off values were determined based on previous reports [9,25,28] and the frequency distribution histograms of these data.

### Computed Tomography Images

Patients underwent dual phase contrast-enhanced computed tomography (CT) imaging prior to treatment. Post-contrast portal venous CT images were obtained following the administration of 150 mL iodinated contrast (Omnipaque 300, GE Healthcare, New Jersey) at 4.0 mL/sec, on multidetector CT (Lightspeed 16 and VCT, GE Healthcare, Wisconsin). The following scan parameters were used: pitch/table speed = 0.938–0.984/9.37–39.37 mm; autoMA 220–380; noise index 12.5–14; rotation time 0.7–0.8 ms; scan delay 40 s after hepatic arterial phase, which is determined by Smart Prep with a region of interest placement in the abdominal aorta at the level of the celiac artery. Axial slices reconstructed at each 2.5 mm interval were used for the analysis. The entire liver was scanned on each CT.

### Image Processing & Quantitative Imaging Phenotype Extraction

Pre-processing of the CT images was undertaken to define the tumor region for further analysis. The tumor region was semi-automatically segmented from neighboring structures using Scout Liver (Pathfinder Technologies Inc, Nashville, TN) [29]. Underlying pixel variations in the tumor volume were quantified using a fully automated, software platform for image processing and texture analysis custom built by a computer scientist (A.L.S.) based on a previously reported system [7]. Three-dimensional models of the tumors, bile ducts, and vessels generated by the Scout software were subtracted from the segmented tumor to generate a CT volume of the tumor region with pixel values expressed in Hounsfield units (HU). Attenuation values outside of 0 and 300 HU (corresponding to non-tumoral regions) were removed from the scans and excluded from analysis. The segmented tumor was scaled using conventional image normalization, which compensates for potential irregularities in the scale of pixel values across image volumes while maintaining the overall shape of the image histogram and visual appearance of individual volumes [30]. The final segmented/normalized image was verified by visual inspection prior to further analyses.

**Fig 1:**
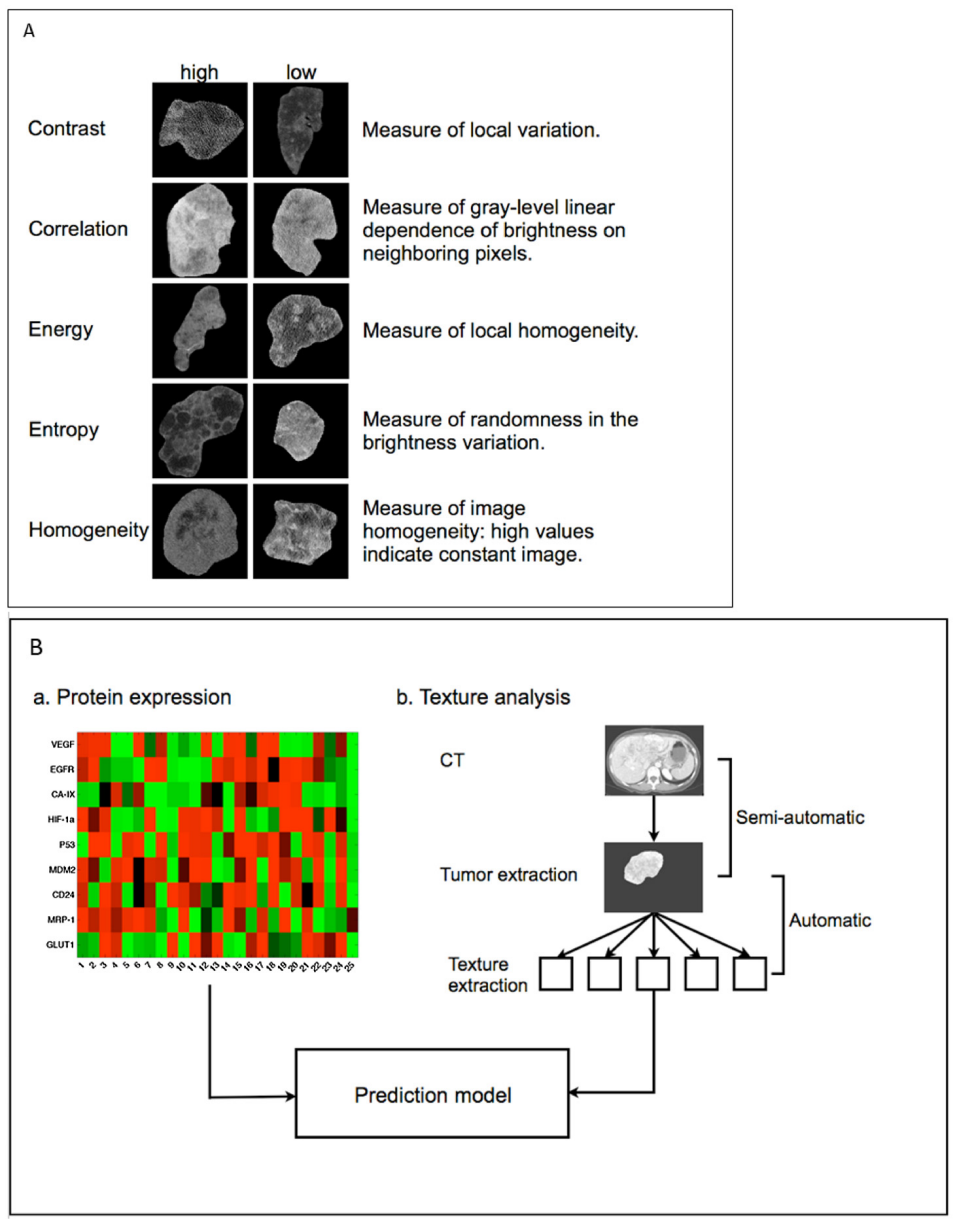
Representative tumors with high and low values for each texture feature (A). Schematic of prediction model of protein expression constructed from quantitative imaging phenotypes. Quantitative image phenotypes are derived via texture analysis: the tumor region is extracted from CT, texture feature statistics are automatically computed based on the region of interest (B).

Second order texture statistics were implemented to examine the spatial relationship of neighboring pixels. Unlike first-order statistics that calculate cumulative statistics on individual pixel values, second-order statistics evaluate the likelihood of observing spatially correlated pixels [31]. A 36 x 36 pixel neighborhood was chosen to assess this spatial relationship because this size demonstrated excellent discriminatory power in preliminary analyses. Gray-level co-occurrence matrices (GLCM) were constructed to represent spatial relationships in the pixel neighborhoods using the standard implementation in the Image Processing Toolbox in MATLAB (MathWorks, Natick, MA). Four GLCM-based texture feature statistics were used: contrast (local variation in the image), correlation (gray-level interdependence of brightness), energy (local homogeneity), and homogeneity (high values indicate constant image). Each were computed in four directions from 0°, 45°, 90°, and 135°. Values were averaged over the four directions since the statistics were found to be directionally invariant. Values were computed for each CT slice and averaged over the whole tumor volume. A fifth texture statistic, image entropy (measure of randomness in brightness variation), was computed for the entire tumor. Tumors with high and low values for each texture feature are illustrated in Fig 1A. Quantitative imaging phenotype was defined as either a single or combination of texture features, which measure tumor heterogeneity.

### Qualitative Imaging Features

Qualitative imaging features (S2 Table), which have been previously reported [2], were evaluated by an attending diagnostic radiologist from the section of Abdominal Imaging (R.K.G.D.), in order to assess whether radiographic inspection alone could predict the relationship between imaging features and protein expression levels.

### Statistical analysis

Descriptive and comparative statistics were performed using Statistical Software for the Social Sciences (SPSS) version 22 (IBM Corporation, Armonk, NY, USA). Continuous variables were compared using the Student t-test or Mann-Whitney test, as appropriate by the type of distribution. Categorical variables were compared using χ2 or the Fisher exact test depending on the number of observations. Survival distributions were estimated using the Kaplan-Meier method and compared using the Cox-regression model. Time to event was calculated from initiation of hepatic artery infusion pump [16,17]. Patients without the event of interest at last follow-up were censored.

Linear regression analysis was undertaken to assess the relationship between protein expression and texture features (both as continuous variables). A regression line with protein expression levels as the dependent variable (y-axis) and texture feature as independent variable was derived and Pearson’s correlation coefficient was calculated for all combinations of texture features and protein expression variables. Confident intervals with α = 0.05 were computed for each regression. A multiple linear regression modeled the relationship between combinations of texture features in an attempt to show the predictive power of texture feature sets (i.e., quantitative imaging phenotypes). A schematic of the study is presented in Fig 1B.

## Results

### Clinicopathologic and molecular characteristics

Forty-four patients with initially unresectable ICC were previously included in the two phase II trials mentioned above. In the current study, we excluded twenty patients due to inadequate tissue for immunohistochemical staining and 3 patients with preoperative CT scan inadequate for texture analysis. Of note, 4 patients who were taken off the previous two trials after the pre-treatment biopsy and hepatic artery infusion pump placement were included in the current study except for the outcome analyses (i.e. response and survival). A total of 25 patients with histologically proven ICC were included in the current study. The median age was 62 years (range: 54–84) and 20 patients (80%) were female. The median tumor size was 10.2 cm (range: 4–14), 10 (40%) were single tumors, and 90% were moderately differentiated. Positive immunohistochemistry staining was recorded for VEGF in 67% of the cases, EGFR in 75%, CD24 in 55%, CA-IX in 92%, HIF-1α in 78%, P53 in 28%, MDM2 in 20%, MRP-1 in 22%, and GLUT1 in 52% (S1 Table).

At a median follow-up time of 39 months (range: 11–90 months) the median overall survival (OS) was 37 months (95%CI: 15–59). The median progression-free survival (PFS) was 9 months (95%CI: 6–12) and the response rates were 62%, 33%, and 5% for partial response, stable disease, and progressive disease, respectively. Ultimately, two patients responded sufficiently to undergo resection.

### Quantitative imaging phenotypes, qualitative imaging features, and protein expression levels

**Fig 2:**
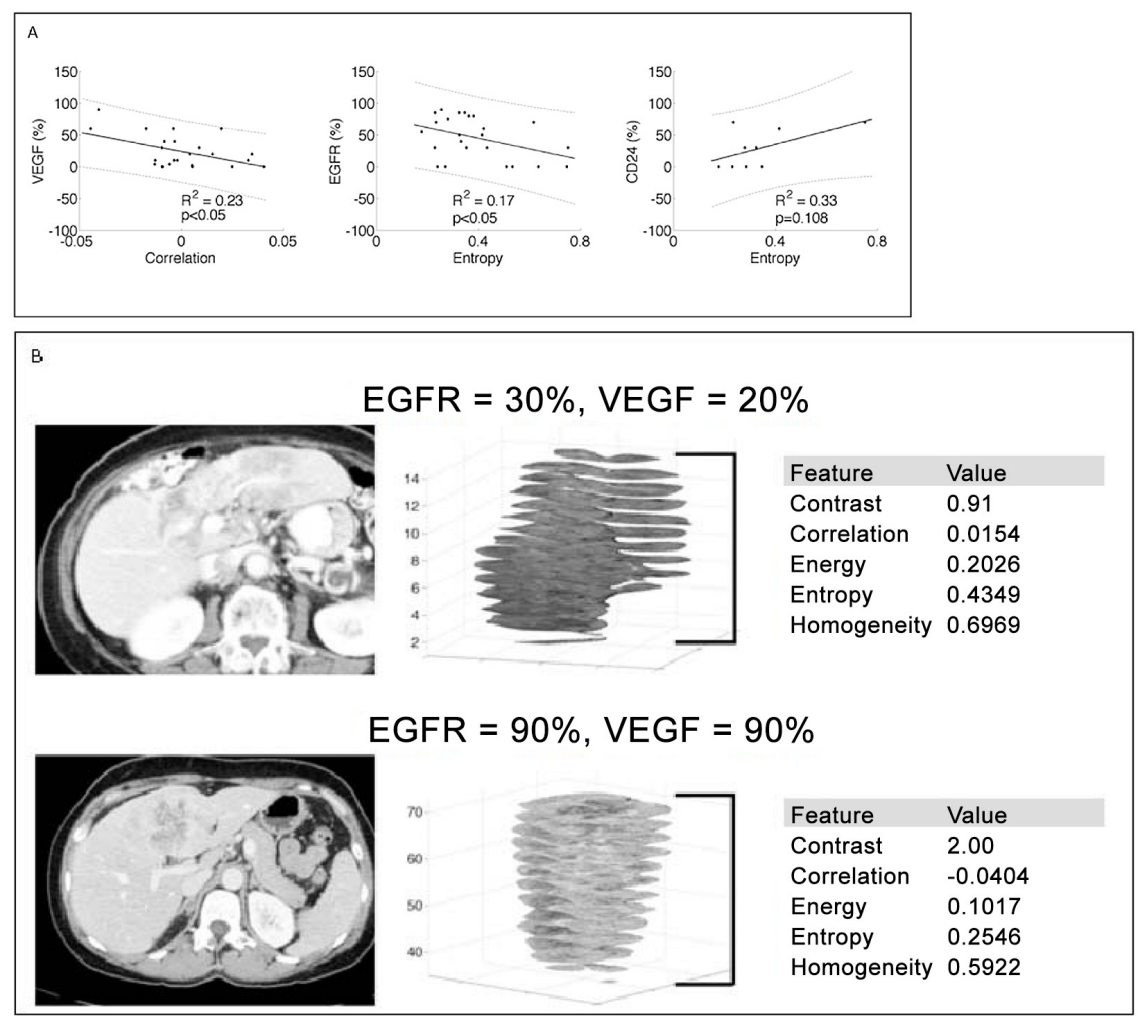
Selected linear regression plots of texture features with respect to protein expression levels. The 95% confidence interval is rendered. For descriptive purpose, each protein is plotted against the texture feature that contributed the most to the prediction model (A). Two intrahepatic cholangiocarcinomas with low (top row) and high (bottom row) VEGF/EGFR protein expression by immunohistochemistry. After semi-automated segmentation of tumor borders from CT images, the tumor pixel attenuation values are evaluated for texture features. Axial slices of the segmented tumors are shown with texture features calculated for each slice and averaged. (B).

Selected linear regression plots of tumor texture features with respect to protein expression levels are shown in Fig 2. Correlation texture feature (R^2^ = 0.23, *p*<0.05) was significantly associated with VEGF expression. Correlation (R^2^ = 0.21, *p*<0.05) and entropy (R^2^ = 0.17, *p*<0.05) texture features were significantly related to EGFR expression. A trend was demonstrated between entropy and CD24 expression (R^2^ = 0.33, *p* = 0.108). By contrast, there were no significant associations between any of the texture features and either CA-IX, HIF-1α, P53, MDM2, MRP-1, or GLUT1 (S3 Table and S1 Fig).

Multiple linear regressions of the significant texture features from the univariate analysis test the predictive power of quantitative imaging phenotypes (i.e., texture features sets). Table 1 summarizes these results. Correlation and contrast explain 26% of VEGF expression (R^2^ = 0.26, *p*<0.05) in this model. In the case of EGFR expression, discriminatory power of the model was substantially improved when all five texture features were combined (R^2^ = 0.47, *p*<0.05) with the combination of correlation, entropy, and homogeneity representing a substantial portion (R^2^ = 0.41, *p*<0.05). A trend was observed between CD24 expression levels and the combination of entropy, correlation, and homogeneity (R^2^ = 0.68, *p* = 0.1). Fig 2B compares representative texture feature values for tumors with low and high VEGF and EGFR expression. No association was noted between outcome variables (OS, PFS, or time to progression) and either VEGF, EGFR, or CD24 expression levels (data not shown).

**Table 1:** Multiple linear regression analysis of hypoxia markers and quantitative imaging phenotypes.

Hypoxia markers (%)	Imaging phenotype	R^2^	*p*-value
**VEGF**	Entropy, energy, correlation, contrast, homogeneity	0.3	0.2
	Entropy, energy, correlation, contrast	0.3	0.12
	Entropy, correlation, contrast,	0.28	0.08
	Correlation, contrast	0.26	**0.04**
	Correlation	0.23	**0.016**
**EGFR**	Entropy, energy, correlation, contrast, homogeneity	0.47	**0.029**
	Entropy, energy, correlation, homogeneity	0.43	**0.025**
	Entropy, correlation, homogeneity	0.41	**0.013**
**CD24**	Entropy, correlation, energy, contrast, homogeneity	0.73	0.36
	Entropy, correlation, energy, homogeneity	0.73	0.18
	Entropy, correlation, homogeneity	0.68	0.104

Table 2 details thirteen qualitative imaging features and demonstrates that VEGF expression was associated with two qualitative imaging features (‘tumor-liver difference’ and ‘attenuation heterogeneity’, *p*<0.05 for both). CD24 expression was correlated with ‘biliary dilatation’ (*p*<0.05), whereas EGFR was not associated with any of the quantitative imaging features. Notably, none of these three qualitative imaging features correlated with any of the texture features (data not shown).

**Table 2:** Relationship between qualitative imaging features and protein expression levels by linear regression.

Imaging features^a^	EGFR (%)			VEGF (%)			CD24 (%)		
	β	P-value	R^2^	β	P-value	R^2^	β	P-value	R^2^
**Tumor volume, cc**	-0.17	0.4	0.3	-0.2	0.3	0.2	0.6	0.07	0.4
**Tumor—Liver Difference, Maximum**	-0.09	0.7	0.007	-0.4	**0.04**	0.2	0.4	0.3	0.2
**Attenuation Heterogeneity, Maximum**	-0.09	0.7	0.008	-0.5	**0.02**	0.2	0.3	0.4	0.1
**Internal arteries**	-0.06	0.8	0.004	0.3	0.2	0.07	-0.01	0.9	<0.001
**Capsule**	-0.3	0.1	0.1	-0.05	0.8	0.003	0.3	0.3	0.1
**Hypodense halo**	0.2	0.4	0.03	0.01	0.9	<0.001	NA	NA	NA
**Wash out**	0.3	0.1	0.1	-0.05	0.8	0.003	-0.5	0.1	0.3
**Internal septa**	-0.3	0.1	0.1	0.01	0.9	<0.001	0.3	0.4	0.1
**Tumor margin score max**	0.3	0.2	0.07	-0.1	0.6	0.01	-0.1	0.7	0.02
**Liver capsule abutment**	0.3	0.2	0.09	0.1	0.6	0.01	NA	NA	NA
**Liver capsule buldge**	0.1	0.5	0.02	0.1	0.6	0.01	NA	NA	NA
**Capsule retraction**	0.1	0.7	0.009	-0.1	0.5	0.02	-0.5	0.2	0.2
**Biliary dilatation**	-0.1	0.5	0.02	-0.3	0.1	0.1	0.7	**0.048**	0.5

NA–not analyzed.

^a^Imaging features are defined in S2 Table.

## Discussion

The current study demonstrates a link between imaging phenotypes and molecular profiling of tumors using a cholangiocarcinoma model. ICC exhibits a high degree of abnormal tissue vasculature [14] and expression of hypoxia markers [9]. These features, combined with their large size, make ICC a good model to test the hypothesis that overexpression of hypoxia markers, reflecting a hypoxic microenvironment and relative hypoperfusion, can be detected by high-resolution texture analysis of pixel intensity variation on CT.

Molecular profiling of cancer has become an integral part of treatment selection and provides predictive and prognostic information, as shown in breast cancer by the immunohistochemical assessment of molecular markers such as estrogen receptor, progesterone receptor, and HER2 (human epidermal growth factor receptor 2) [32]. Similar progress has been seen in leukemia, lymphoma, and other malignancies [33]. Molecular profiling methods require invasive tissue procurement procedures with inherent risks (i.e., pain, infection, bleeding, or cancer seeding). In addition, these methods are limited in their utility, since the tissue obtained is necessarily sacrificed to extract nucleic acids or proteins for analysis, and can only provide information at a single time point. These aspects represent impediments to their clinical implementation; the ability of imaging data to provide reliable data on clinically relevant molecular markers would, therefore, represent a major advance.

Radiogenomics is an emerging field, which utilizes non-invasive methods to characterize imaging phenotypes and correlate them with the molecular features of tumors [1]. Segal et al. [2] demonstrated the potential of non-invasive imaging to decode the molecular makeup of human liver cancer. Qualitative imaging features, visually assessed by a radiologist, were correlated with gene expression modules (groups of genes with coherent variation in expression across multiple samples). For instance, a combination of 3 imaging features correlated with the expression level of a module, which was highly enriched for genes involved in cell proliferation, including VEGF. However, it is uncertain how the association with gene expression modules will have direct implementation in daily practice.

The current study aimed to identify a relationship between clinically-oriented molecular markers and objective imaging phenotypes. A substantial portion of tumors in this analysis demonstrated positive staining for VEGF (67%), EGFR (75%), and CD24 (55%), as noted in prior reports [9,28]. These proteins and other hypoxia markers are associated with tumor angiogenesis, which results in abnormal vascular beds, characterized by high permeability, high interstitial pressure, and hypoperfusion [15]. Thus, the current study sought to find imaging phenotypes by texture analysis, which quantify visible variations in enhancement. The results show that quantitative texture features were associated with overexpression of certain hypoxia markers. VEGF and EGFR expression levels were associated with specific quantitative imaging phenotypes, whereas CD24 revealed a promising trend, considering the small sample size. It is likely that these quantitative imaging phenotypes reflect perfusion variations in tumors that overexpress these hypoxia markers (VEGF and EGFR), which mirror the hypoxic microenvironment and abnormal tumor vasculature, as proposed by Gatenby et al. [34]. It is noteworthy that three conventional (qualitative) imaging features correlated with protein expression, including ‘tumor-liver difference’ and ‘attenuation heterogeneity,’ which are subjective assessments of overall tumor enhancement and heterogeneity on a five point scale by a radiologist. These results are in line with a report by Segal et al. [2] who demonstrated a similar relationship between VEGF expression and the qualitative imaging features ‘tumor-liver difference’ and ‘attenuation heterogeneity’. However, neither of these conventional (qualitative) imaging features correlated with the quantitative texture features, thereby emphasizing the lack of redundancy between quantitative and qualitative imaging features. Furthermore, the use of qualitative imaging features is also hampered by potential large interobserver variability, as demonstrated in a recent study on the application of imaging criteria in the diagnosis of hepatocellular carcinoma by a group of radiologists [35].

A wide spectrum of clinical applications exists for radiogenomic methods that can predict overexpression of VEGF, EGFR, or other hypoxia markers. Tumor-related angiogenesis is known to affect local tumor growth and metastasis in a variety of human cancers [36]. On the basis of these findings, antiangiogenic agents were recently developed and incorporated into treatment algorithms of multiple cancers [15]. Bevacizumab (anti-VEGF antibody) and cetuximab (anti-EGFR antibody) are integral portion of the treatment guidelines for colorectal cancer [37] and prediction of treatment response to these agents is currently based on the tumor’s molecular profile [37–39]. Recently, a new VEGF inhibitor, ramucirumab, was FDA approved for treatment of advanced gastric adenocarcinoma. As the armamentarium of signal transduction inhibitors enlarges, it is likely that the clinical implications of radiogenomic profiling will grow. Moreover, increased need for non-invasive radiogenomic profiling is expected as neoadjuvant algorithms increase in frequency, and pre-treatment prediction of response will be routinely incorporated. CD24 is a cell adhesion molecule that was shown to be associated with chemo-resistance capabilities and poor survival in cholangiocarcinoma [40,41]. Furthermore, it was proposed that CD24 may have a role as a new target for directed molecular therapy in cholangiocarcinoma, as decreased tumor cell invasiveness was observed with inhibition of CD24 [40]. In view of the small cohort, it is notable that we observed a trend between specific quantitative imaging phenotypes and CD24 expression levels. These findings merit further research given the proposed role for CD24 in the malignant progression of cholangiocarcinoma.

The current study has some inherent limitations. Despite the prospective patient enrollment and data acquisition, selection factors cannot be absolutely excluded. For instance, excluding patients with inadequate tissue for immunohistochemical staining might have resulted in overemphasizing the relationship between imaging phenotypes and molecular profiling. Similarly, potential intra-tumor heterogeneity has not been evaluated, as a single biopsy was obtained. However, ICC are known as relatively homogenous histologically and this approach represents the limitations of daily clinical practice. In addition, the results obtained in a cholangiocarcinoma model, which is known to exhibit a high degree of abnormal tissue vasculature [14] and expression of hypoxia markers [9], might not be generalizable to other tumors. Nevertheless, the current study is unique in that it utilizes texture-based imaging phenotypes in order to segregate tumors expressing VEGF or EGFR. As mentioned above, the current imaging phenotypes are based on objective and automated quantitative image analysis as opposed to qualitative imaging features assessed visually by a radiologist [35]. Moreover, a certain degree of specificity of our results can be appreciated by the fact that only two hypoxia markers correlated with specific imaging phenotypes. The current pre-validation study is not powered to include multiple testing corrections nor to establish a robust prediction model, which will segregate tumors based on their molecular profile. However, ICC is a rare disease and larger studies will be difficult to undertake. Nevertheless, this study presents proof of principle of the utility of imaging phenotypes for profiling tumors based on their molecular makeup. These data will be useful for informing future validation trials.

In conclusion, the current study suggests that radiogenomic methods may predict protein expression of cholangiocarcinomas. Quantitative imaging phenotypes may be a surrogate marker for the tumor’s molecular makeup and allow for the identification of tumors that express VEGF, EGFR, or CD24, regardless of qualitative imaging features. It seems likely that imaging heterogeneity in tumor enhancement pattern reflect regions of abnormal perfusion related to the hypoxic microenvironment, characterized by overexpression of hypoxia markers. Further investigation into the role of quantitative imaging phenotypes in tumors that express hypoxia markers is warranted as it has a potential to impact on therapy.

## Supporting Information

S1 Fig Selected linear regression plots of texture features with respect to protein expression levels. The 95% confidence interval is rendered.
(DOC)

S1 Table Hypoxia related markers and immunohistochemical staining data. (DOC)

S2 Table Imaging features definitions. (DOC)

S3 Table Relationship between texture features and protein expression levels by linear regression. (DOC)
