# Supplementary material for: Cholangiocarcinoma: Correlation between Molecular Profiling and Imaging Phenotypes
Source: PLoS One. 2015 Jul 24;10(7):e0132953. doi: 10.1371/journal.pone.0132953 (PMC4514866; doi:10.1371/journal.pone.0132953)

**Supplementary data**

**S1 Figure.** Linear regression plots of texture features with respect to protein expression levels.


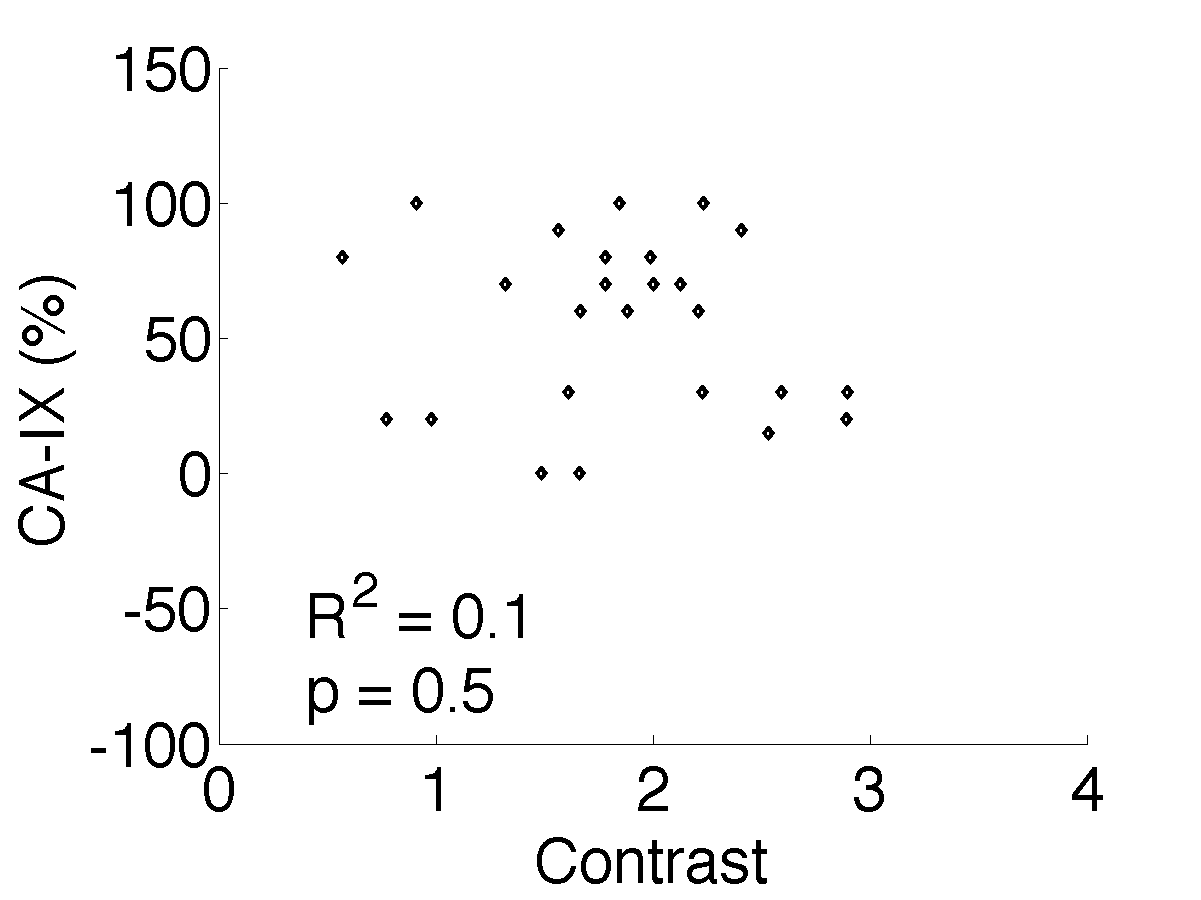


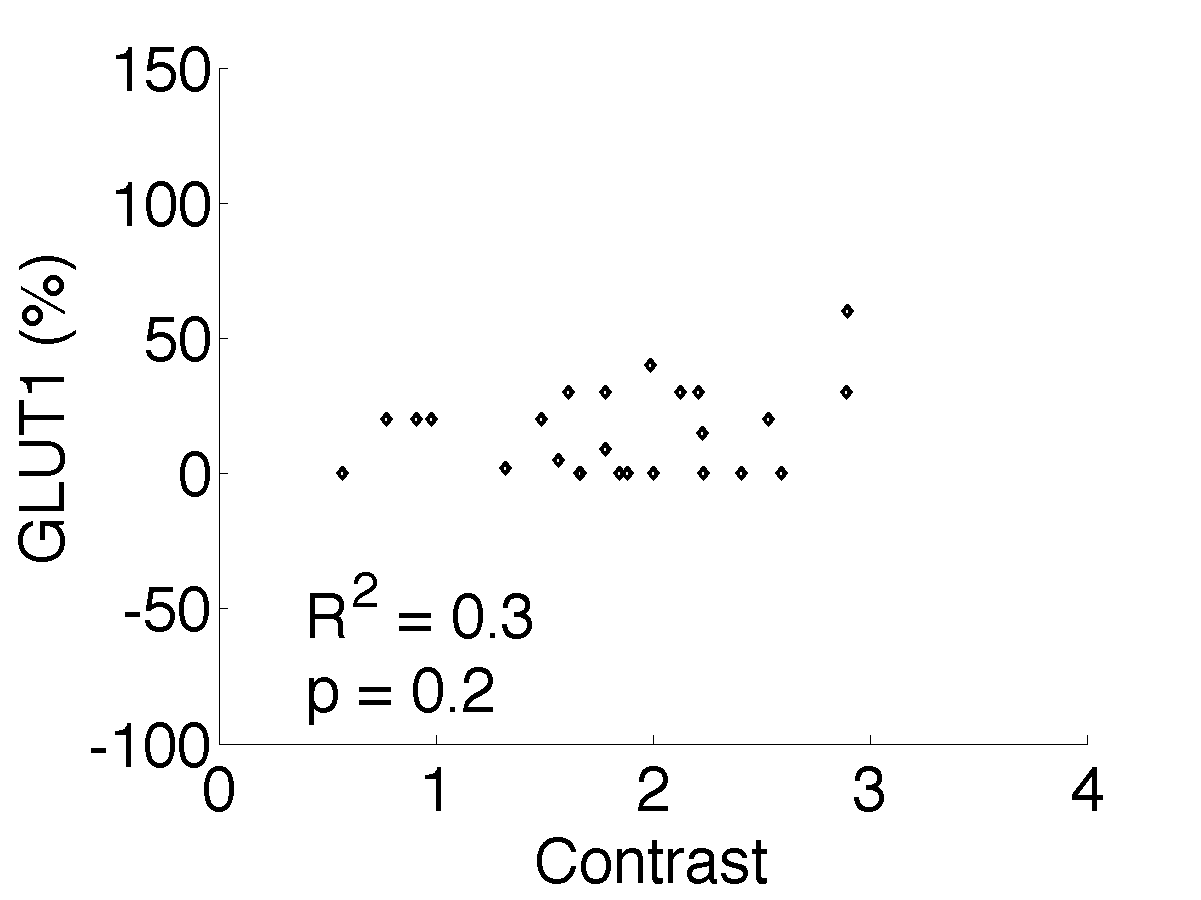


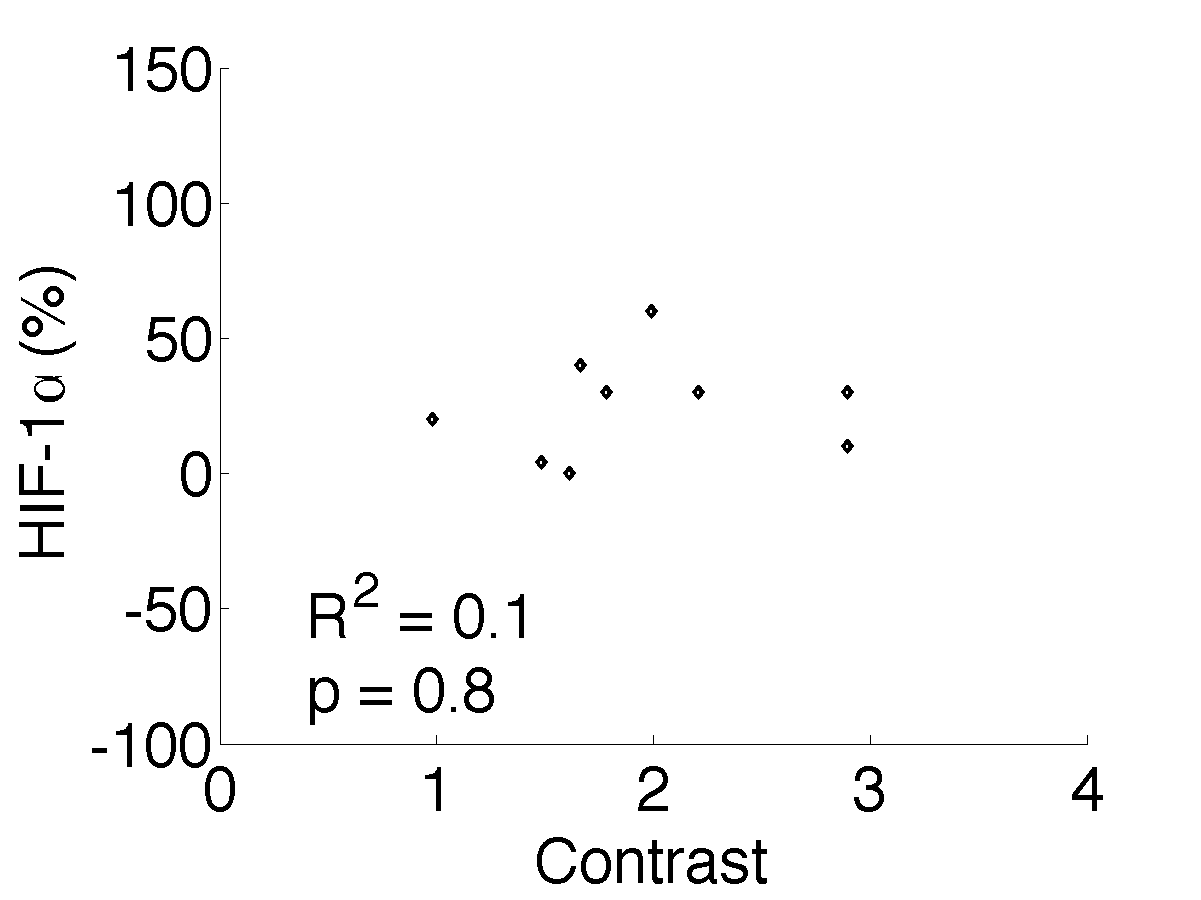


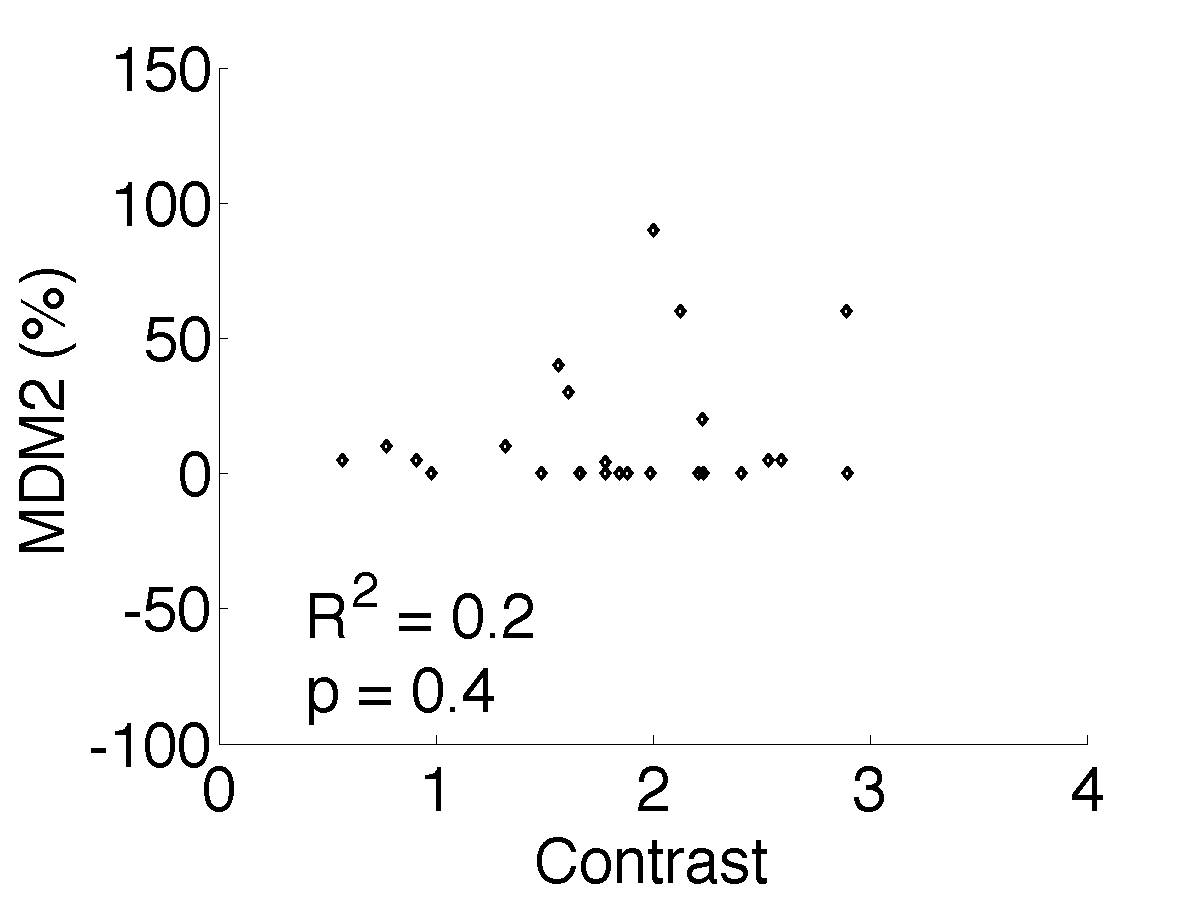


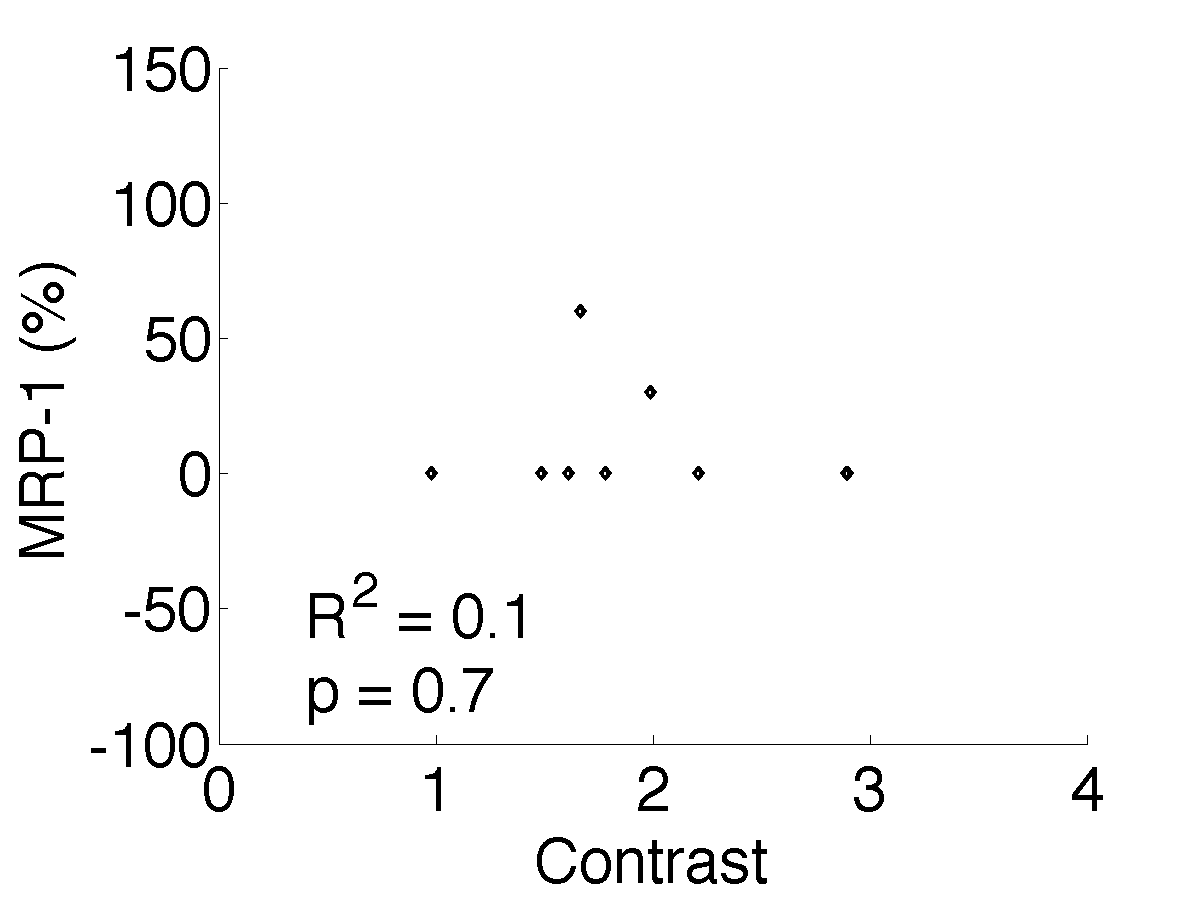


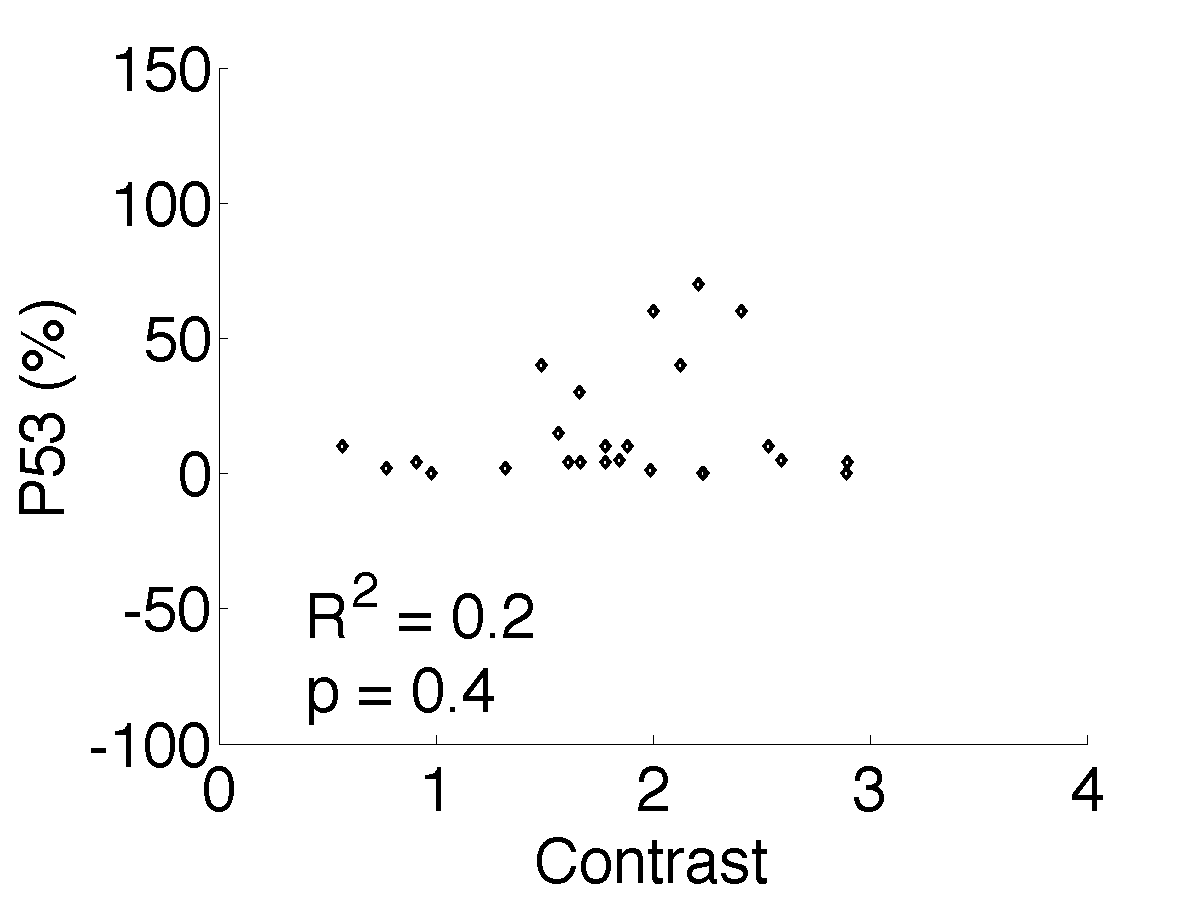


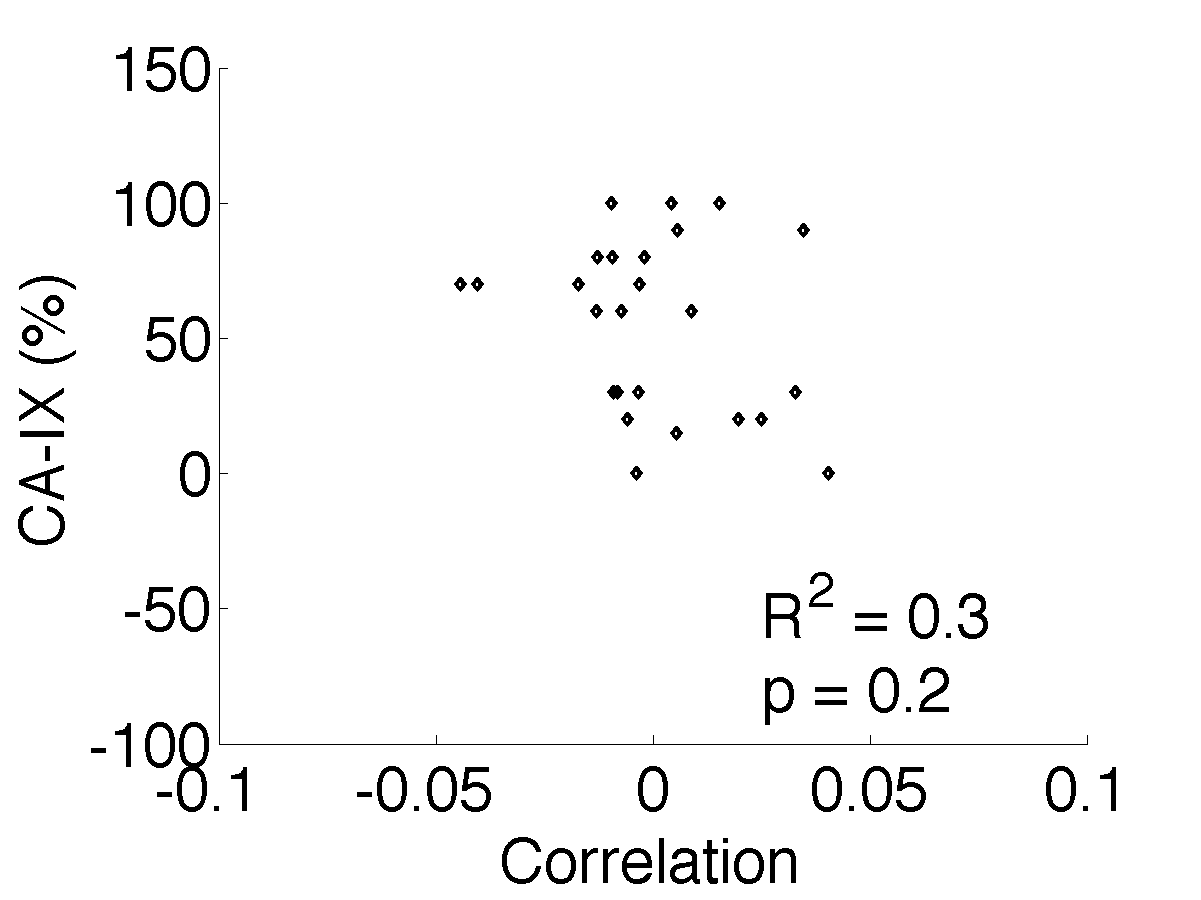


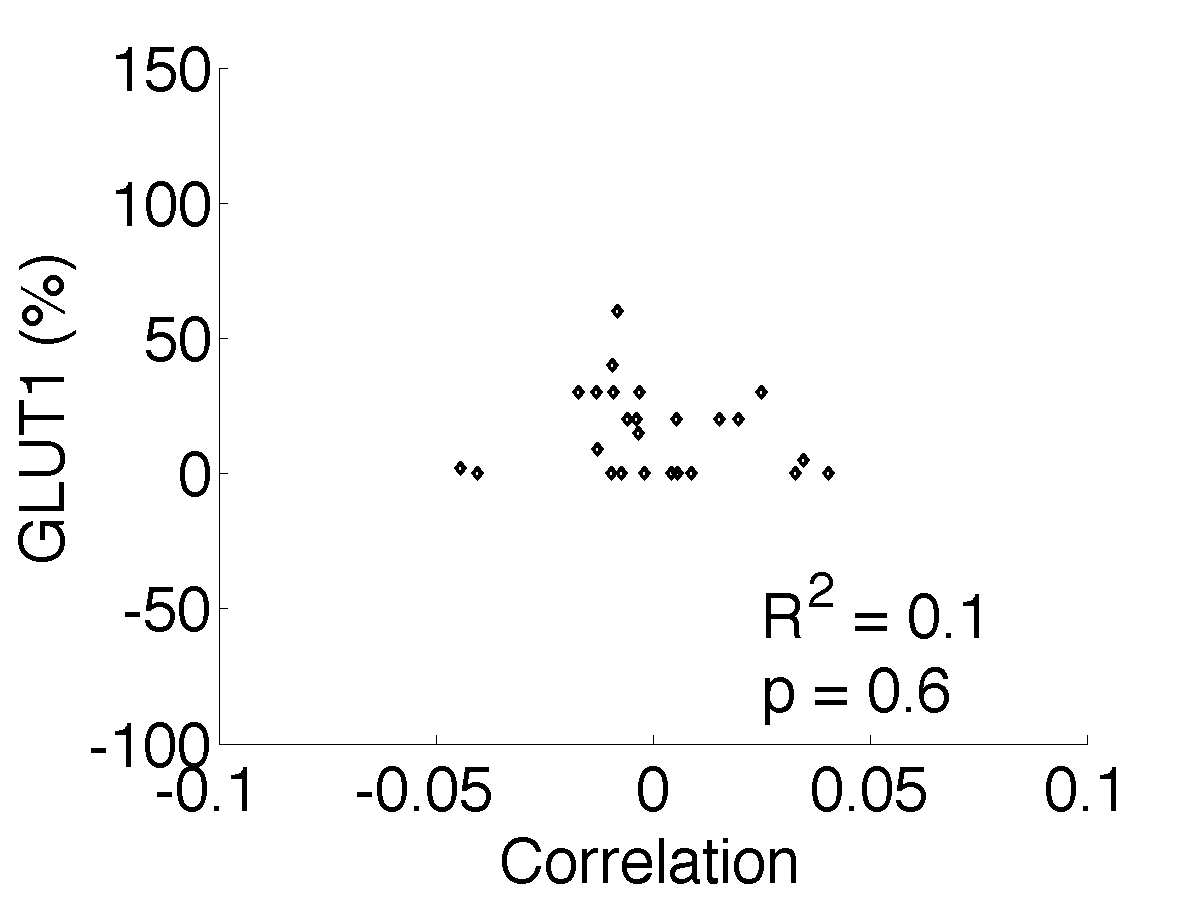


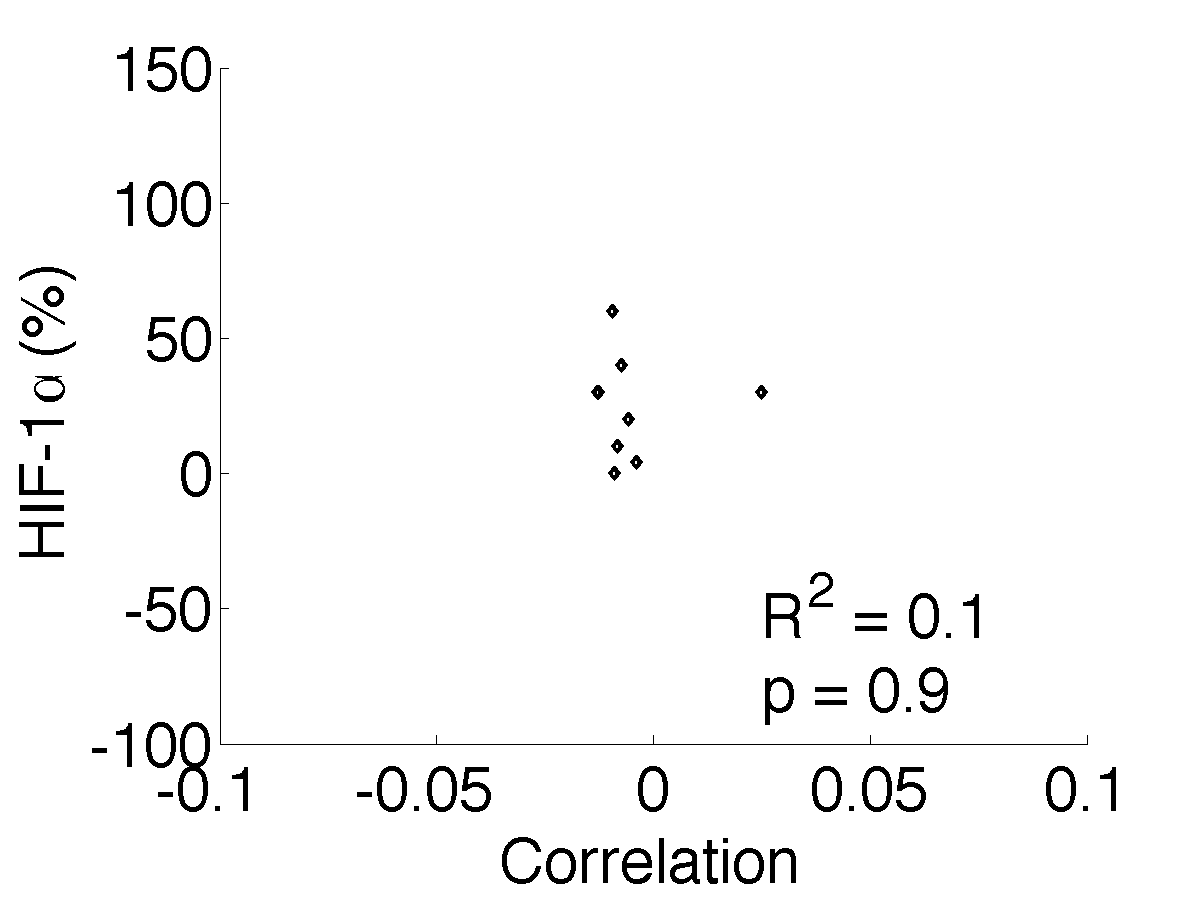


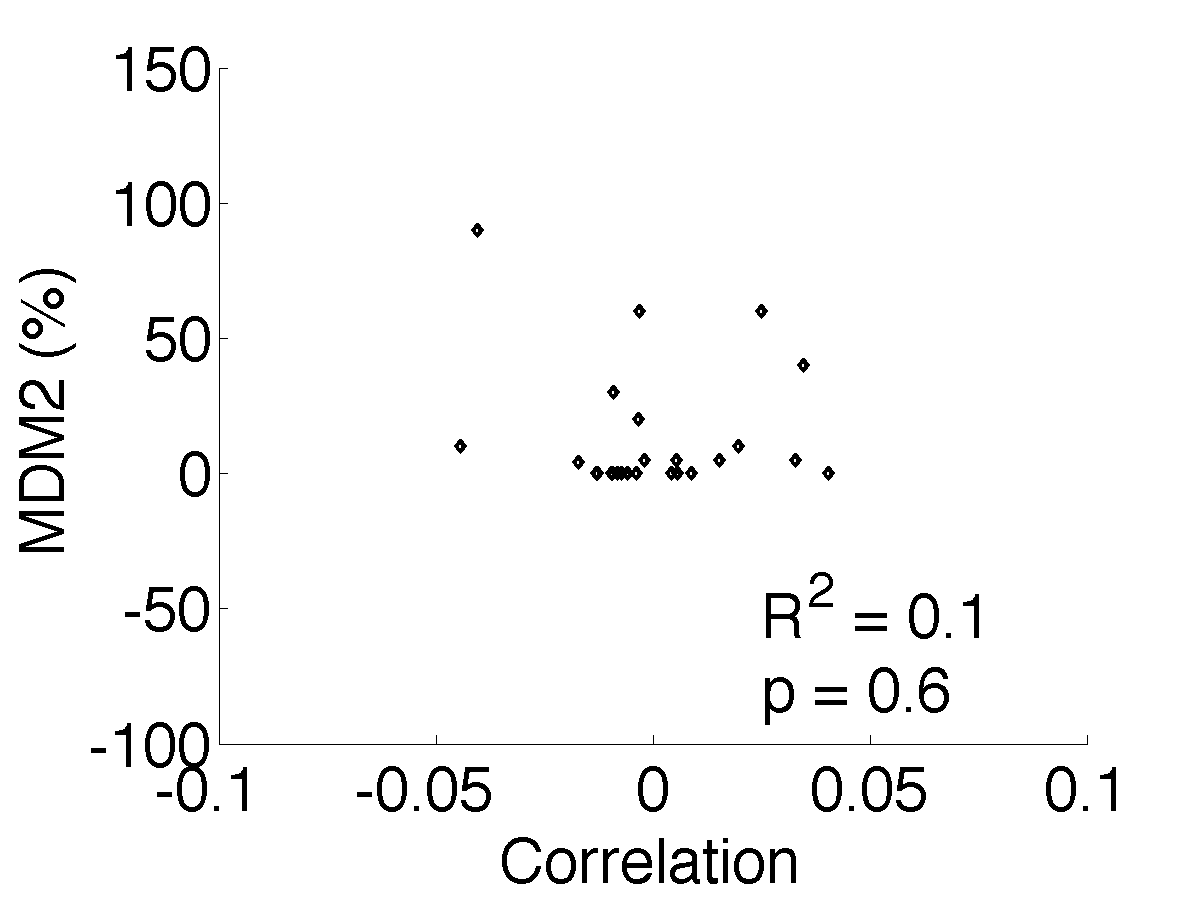


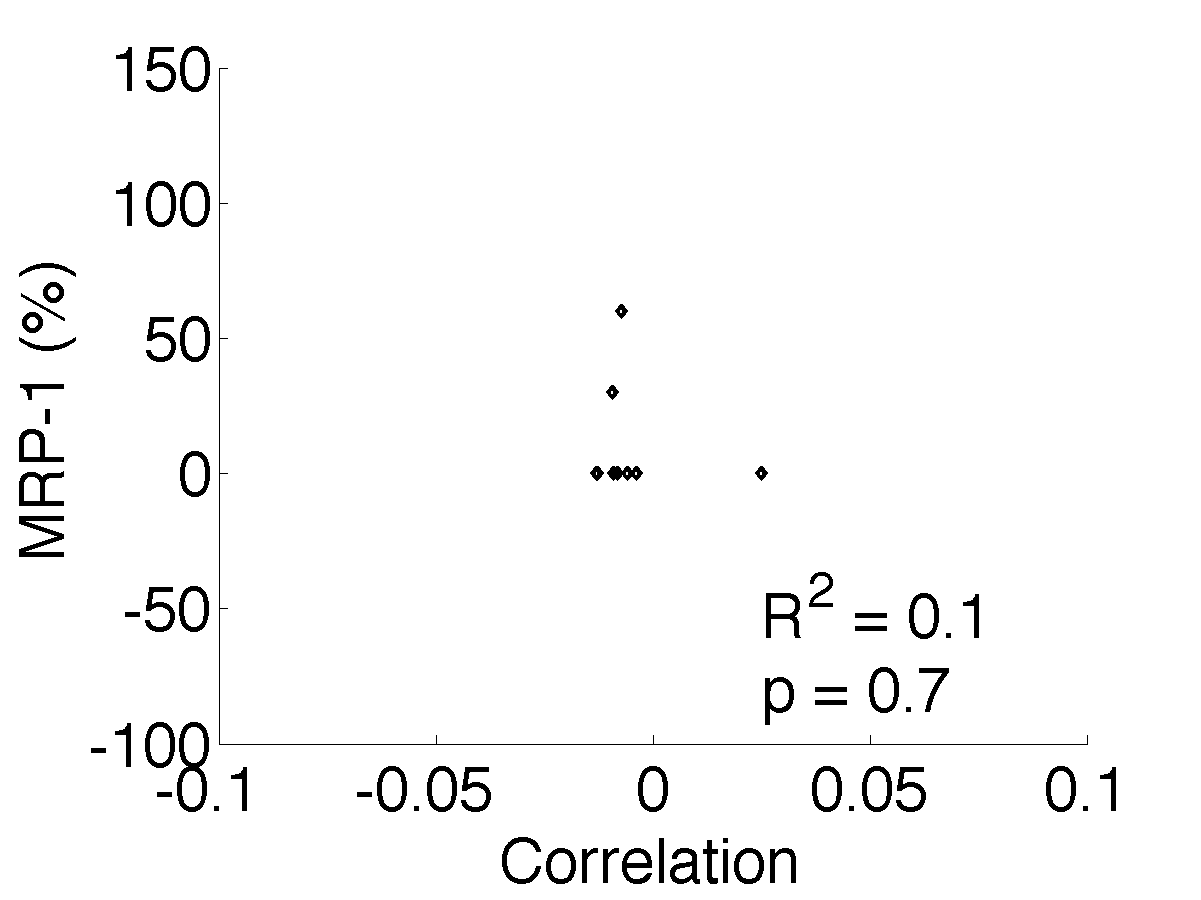


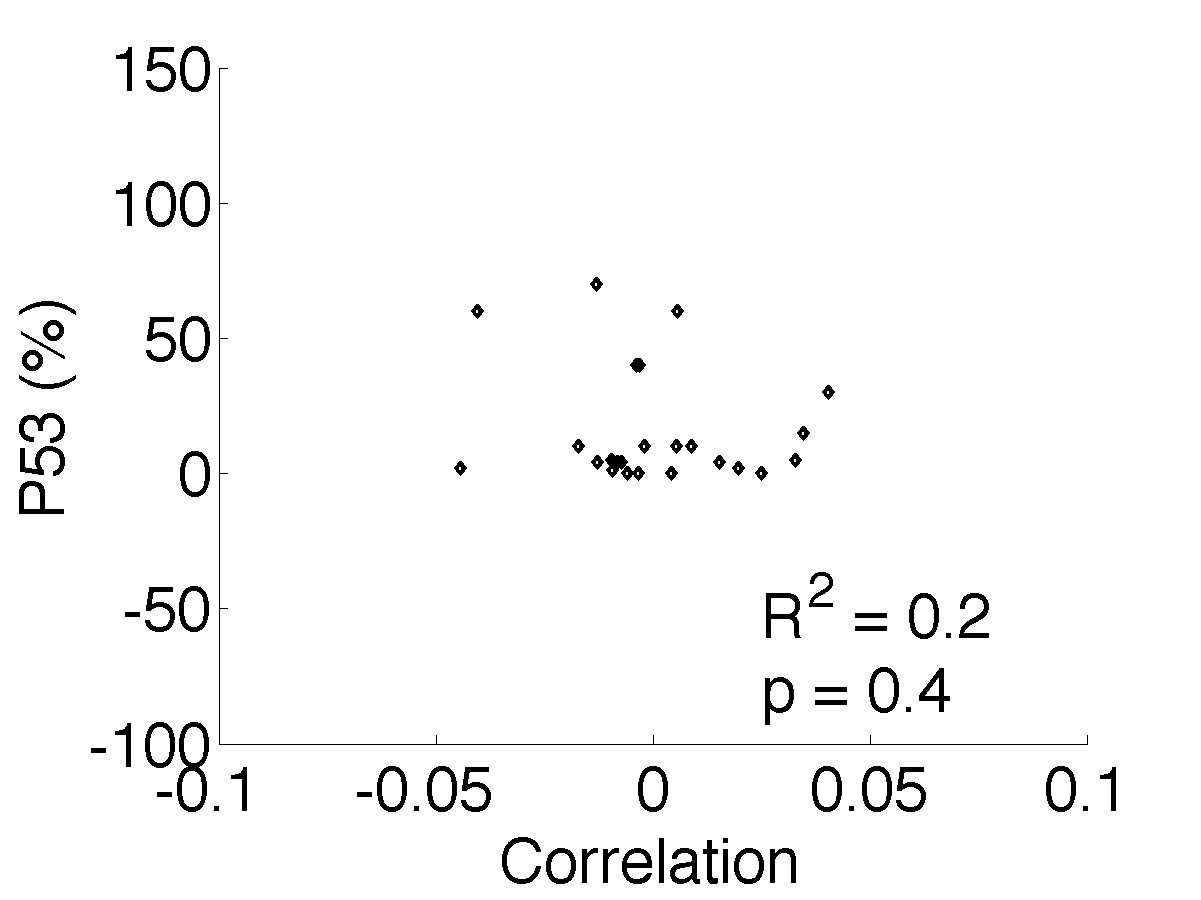


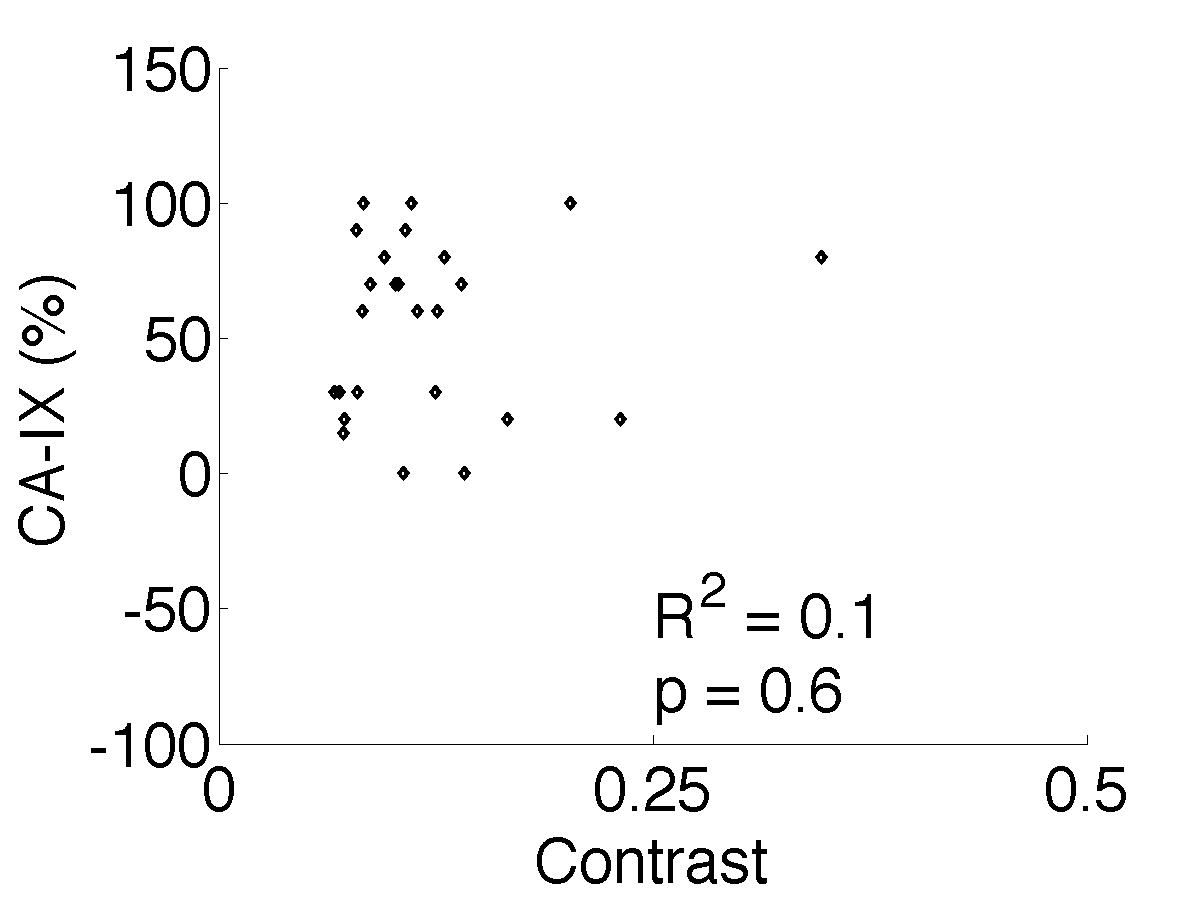


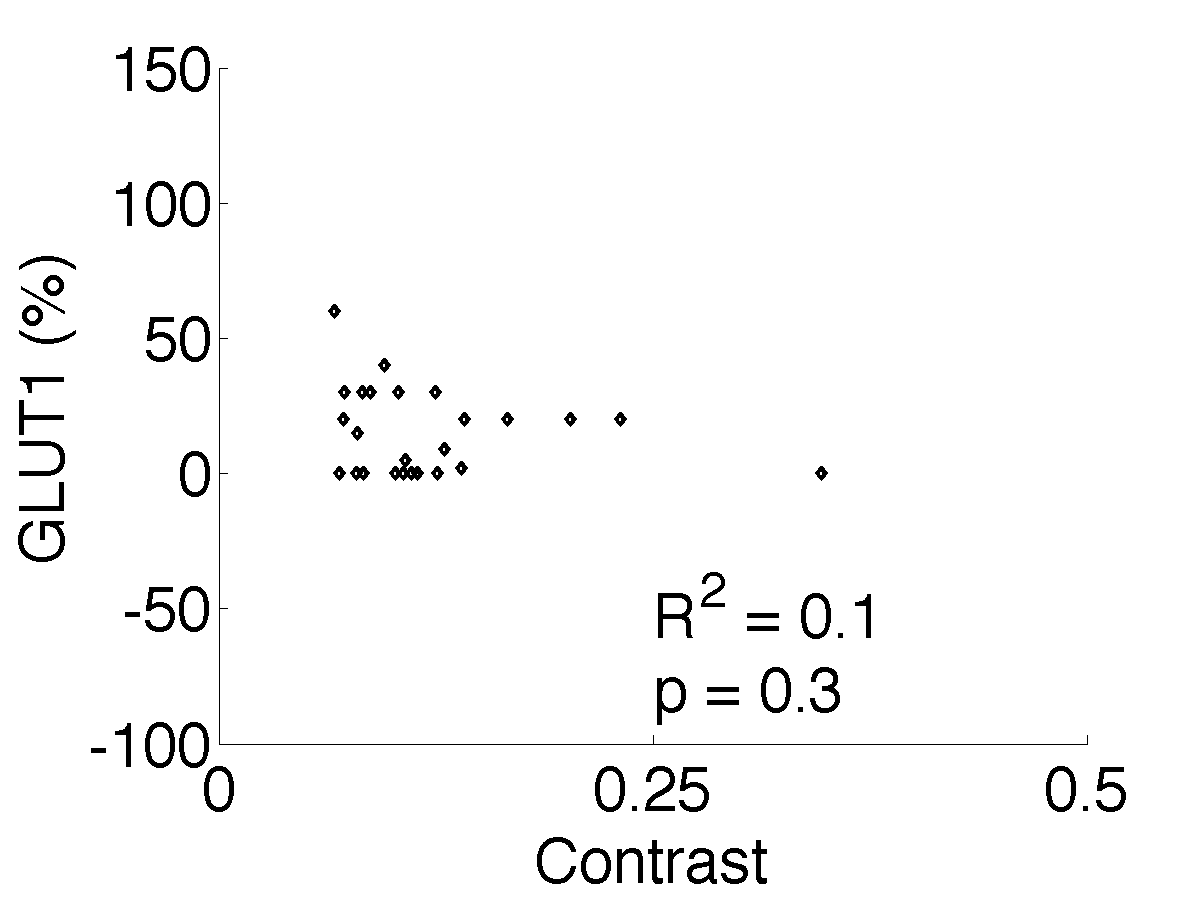


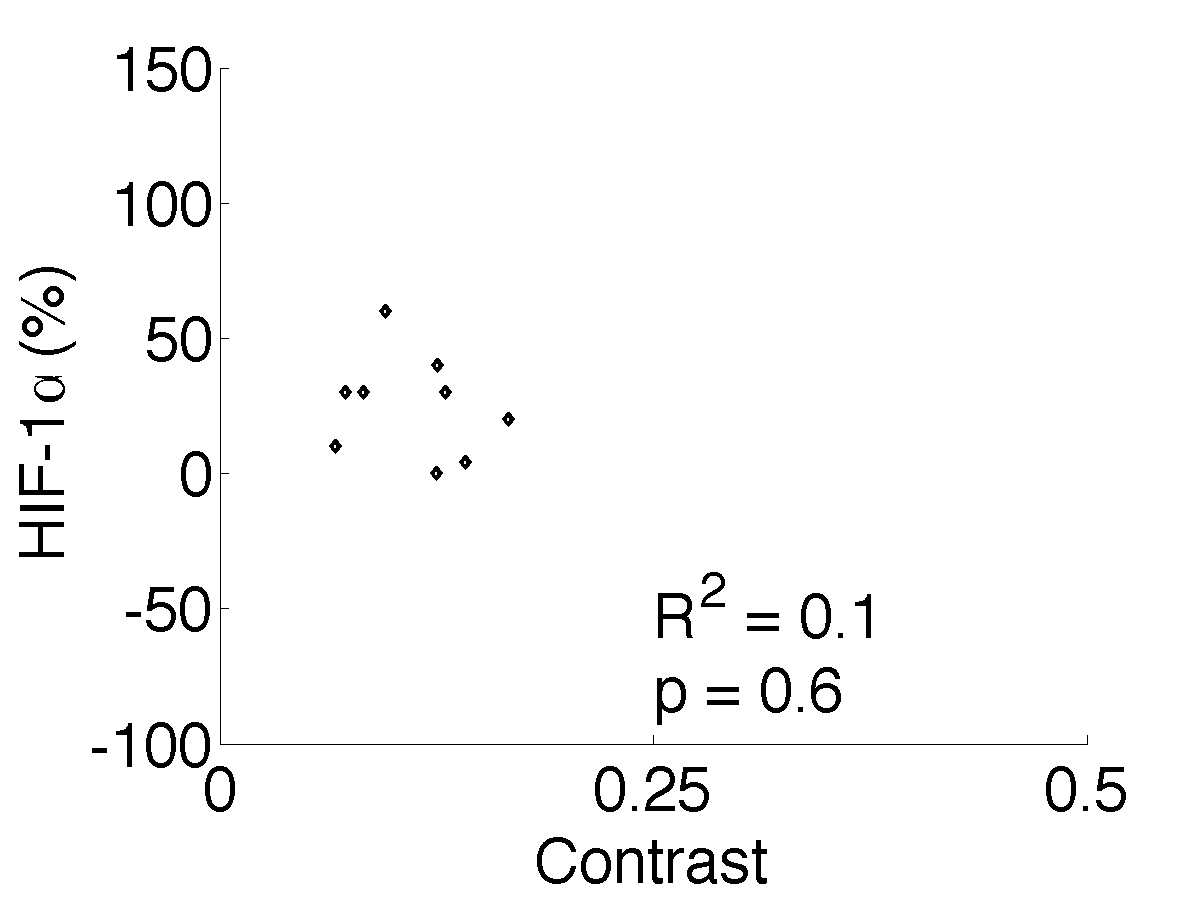


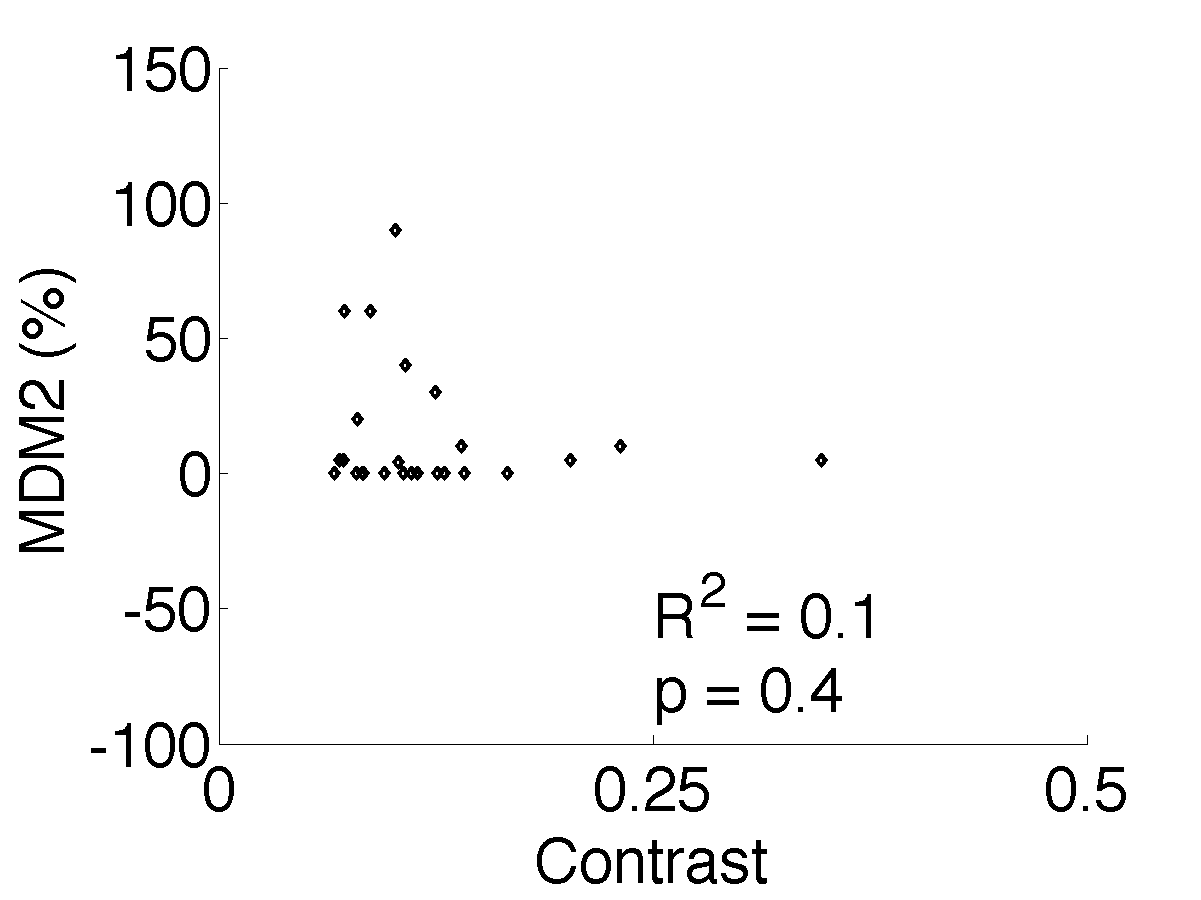


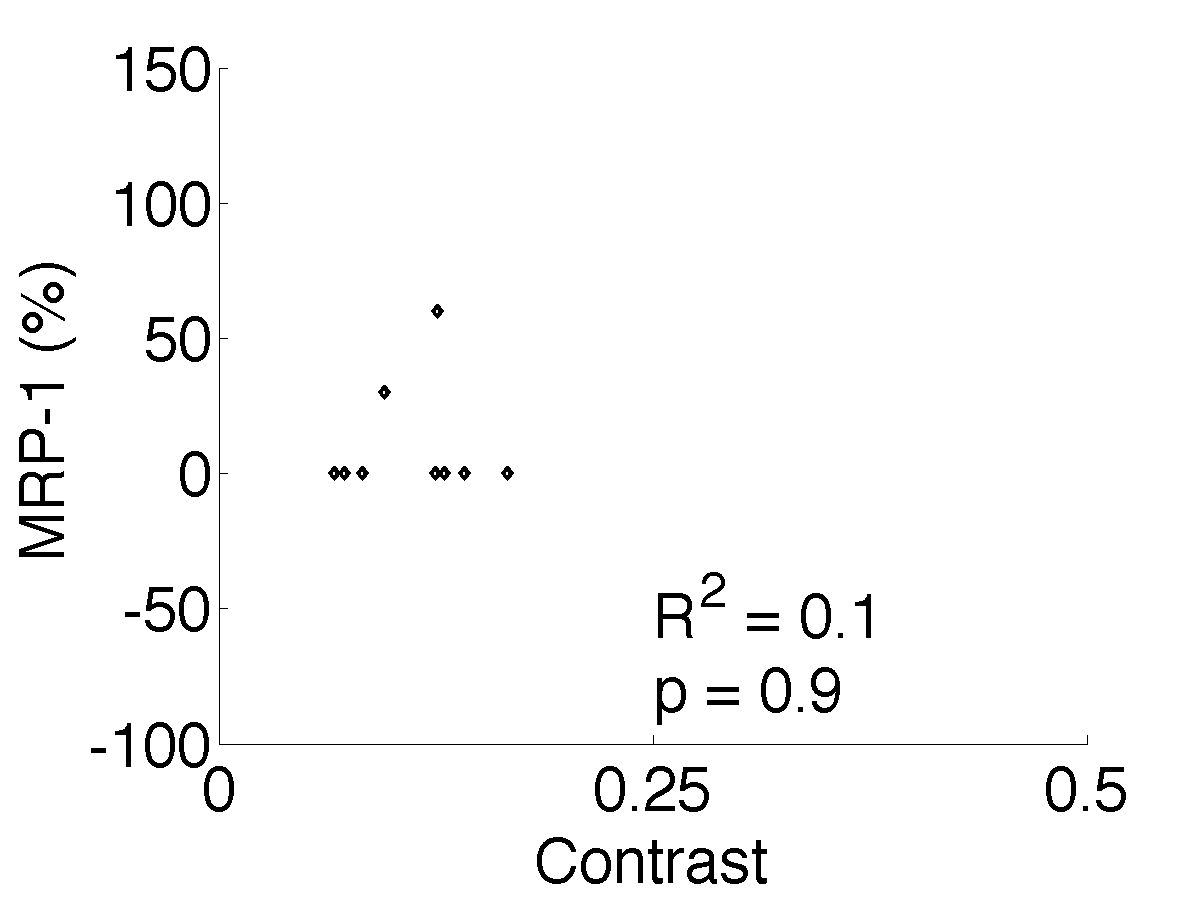


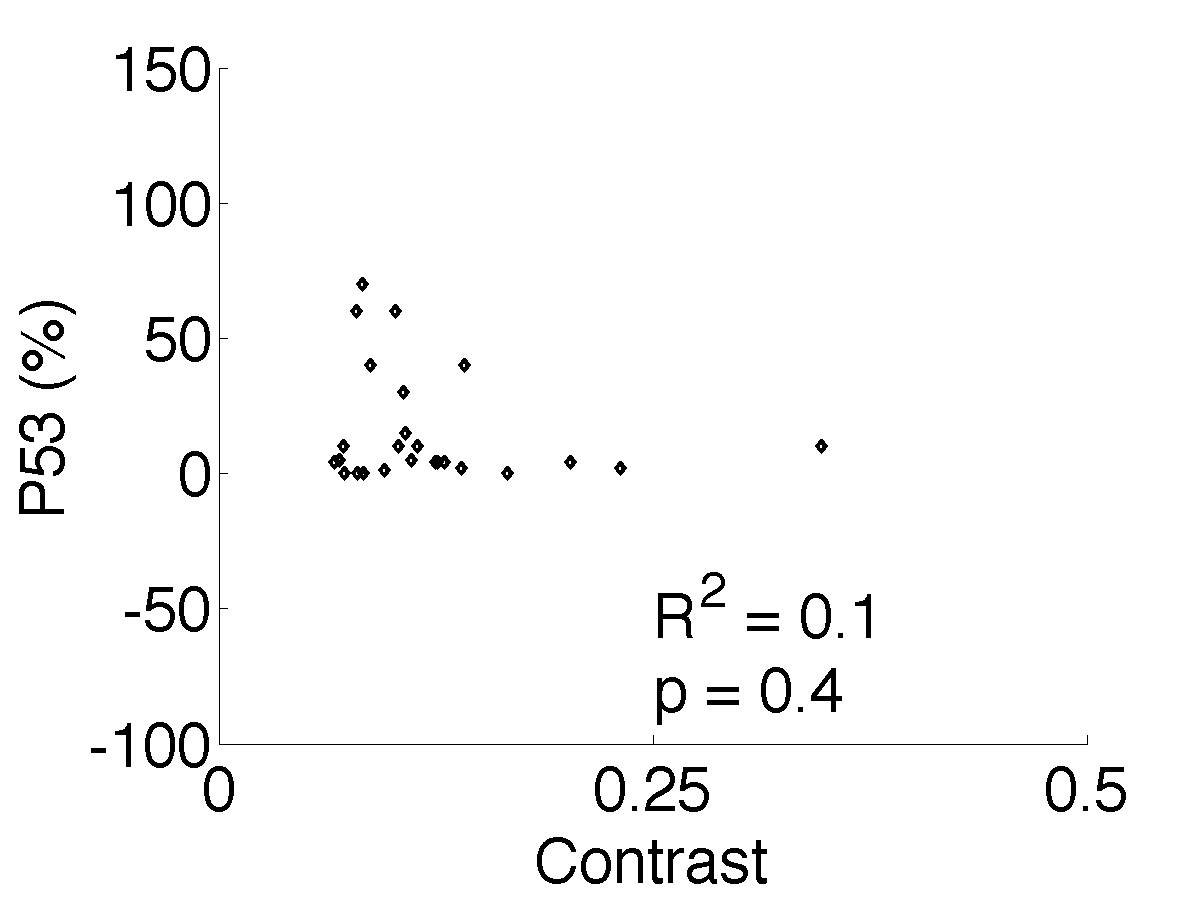


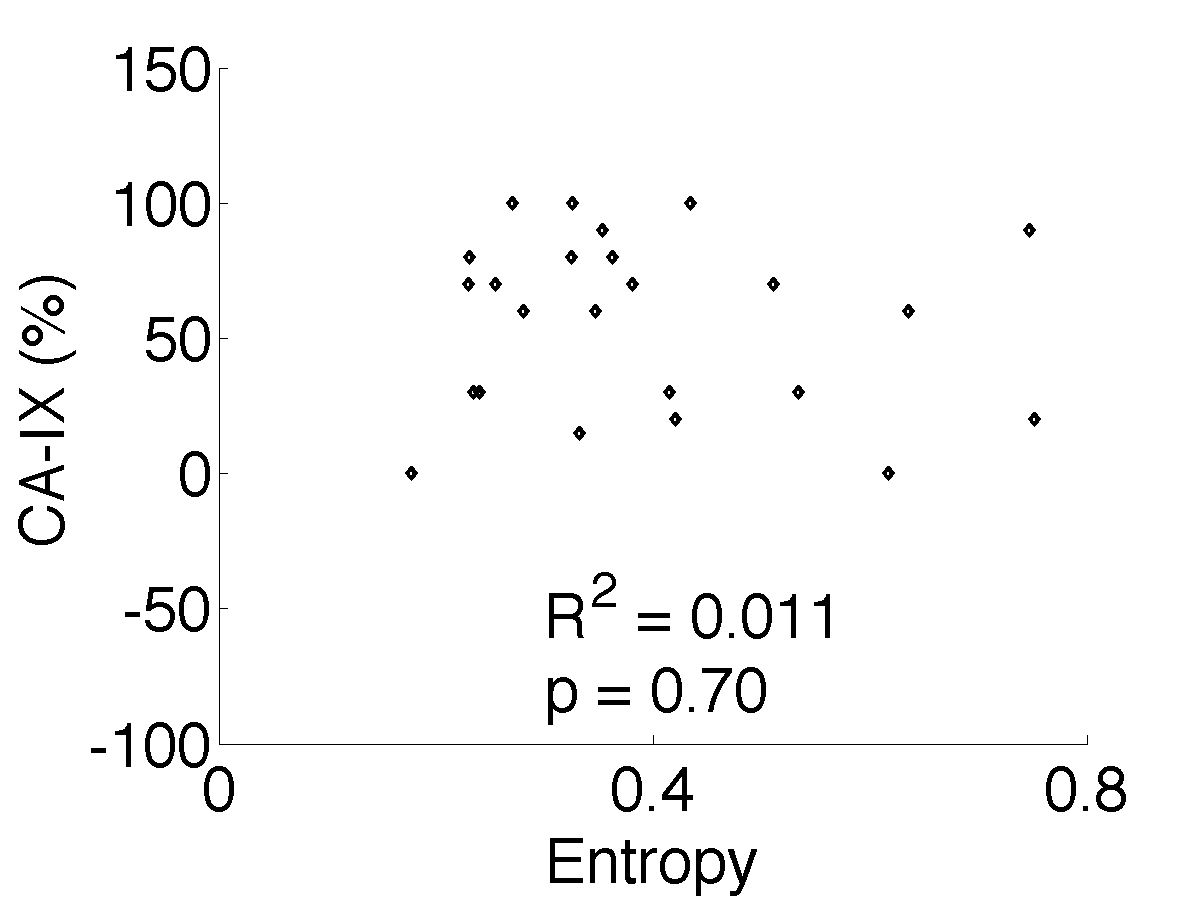


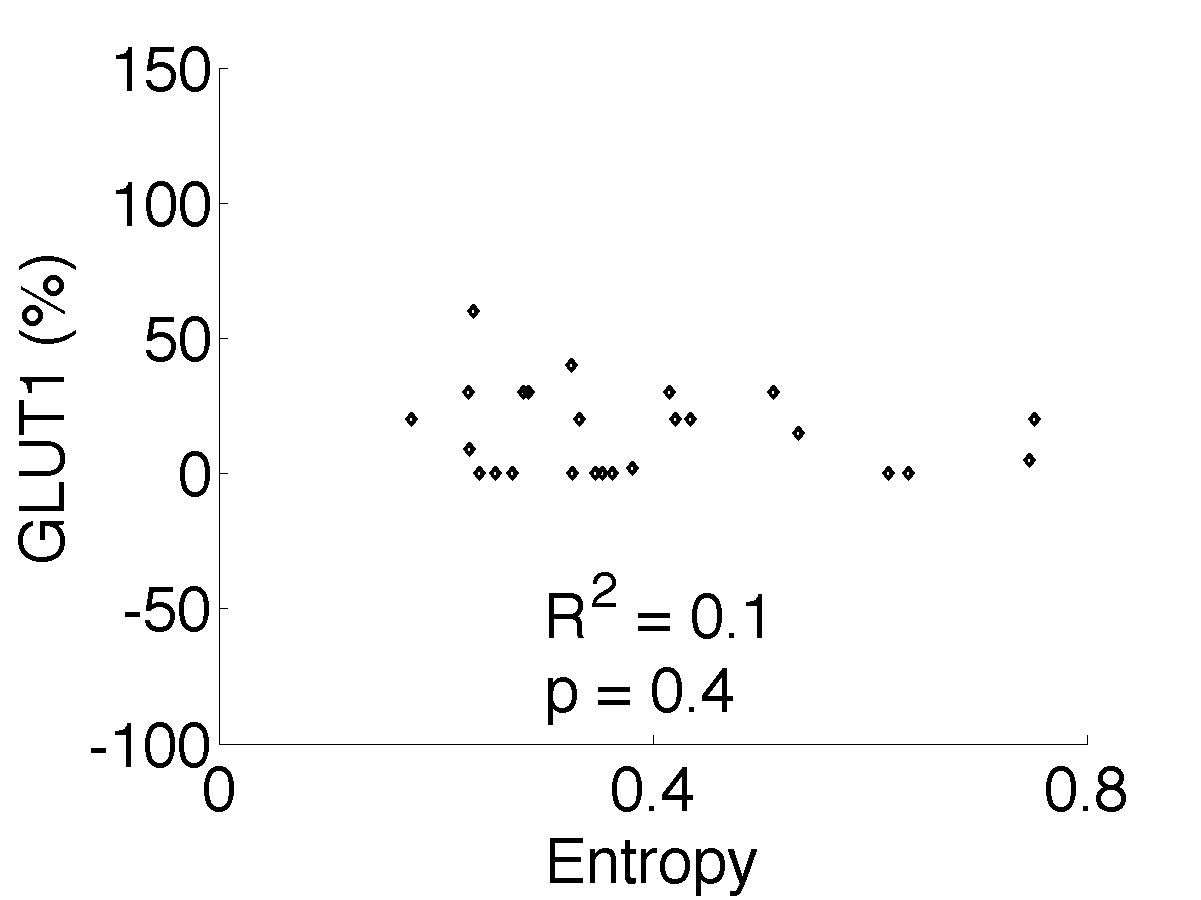


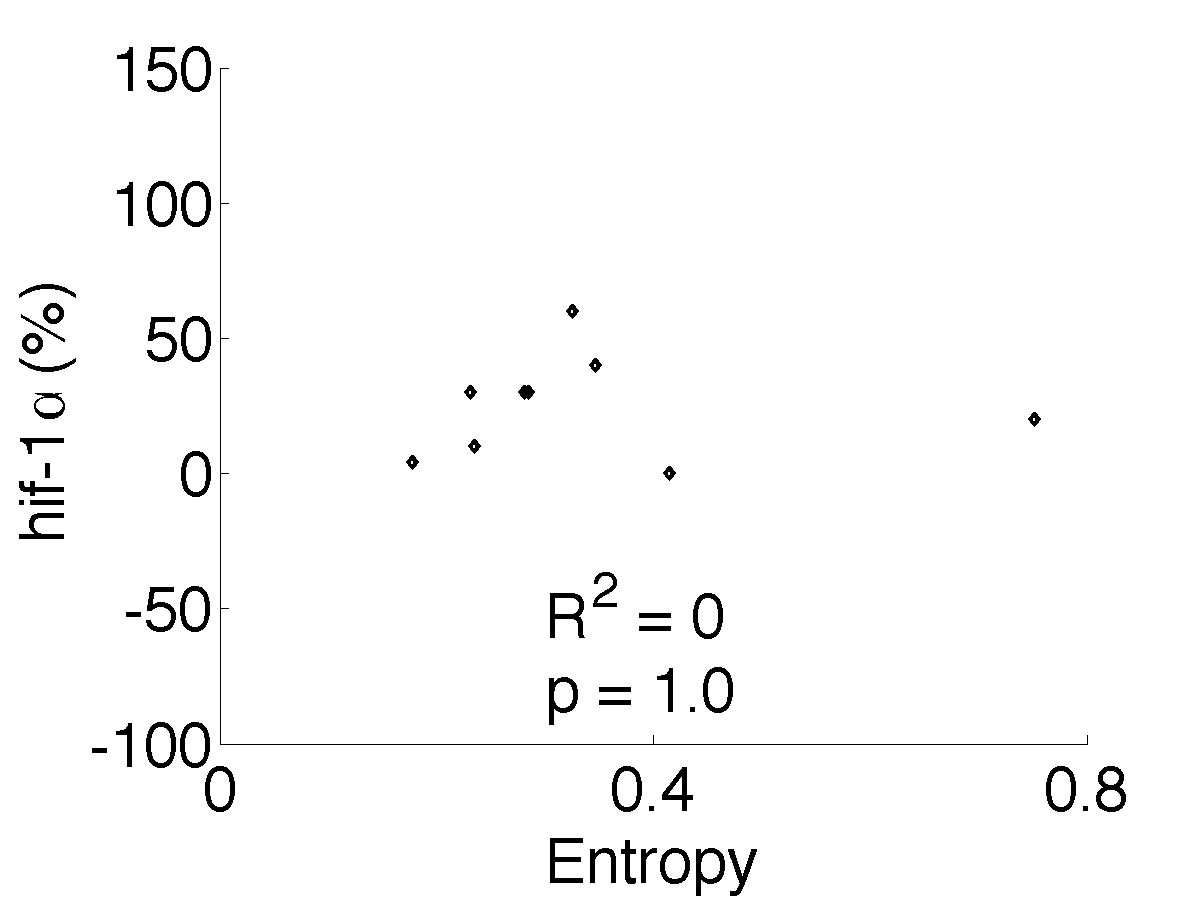


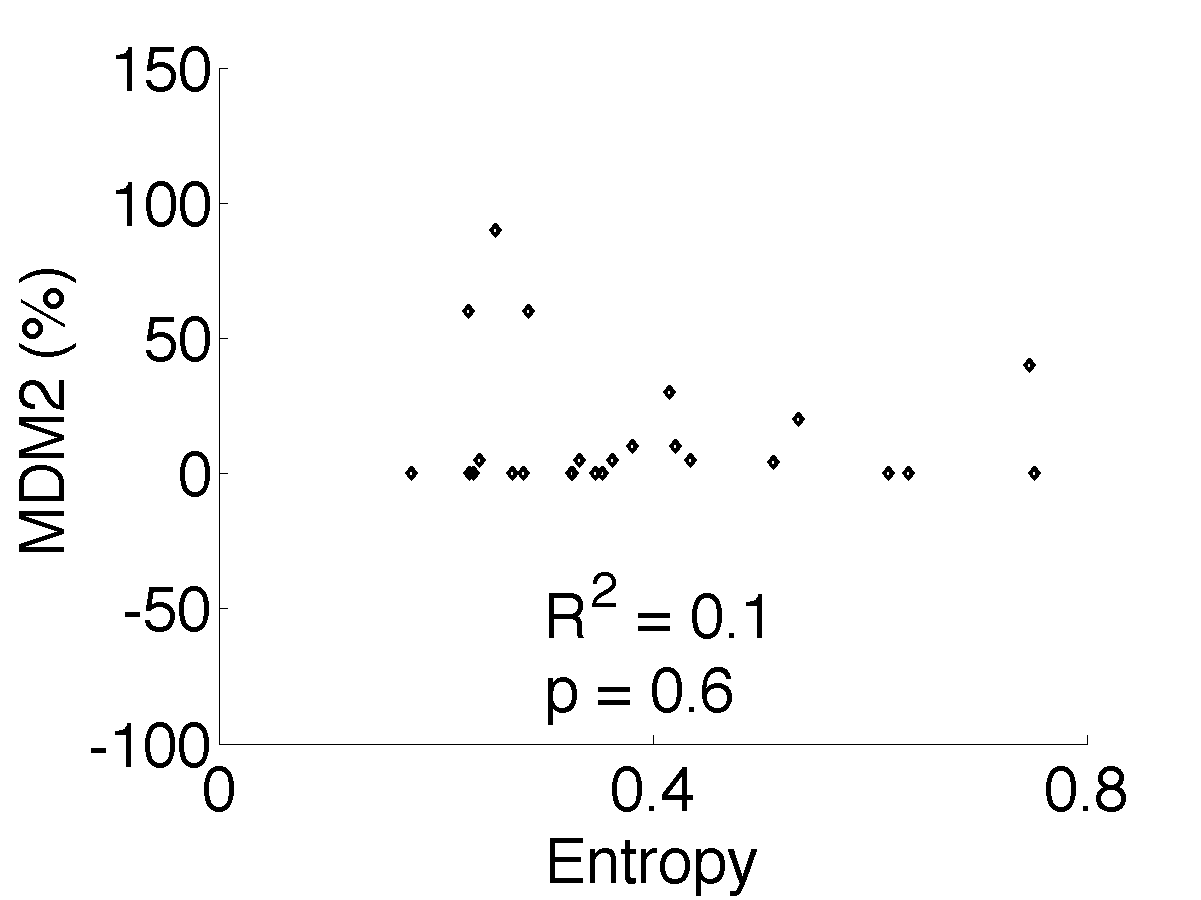


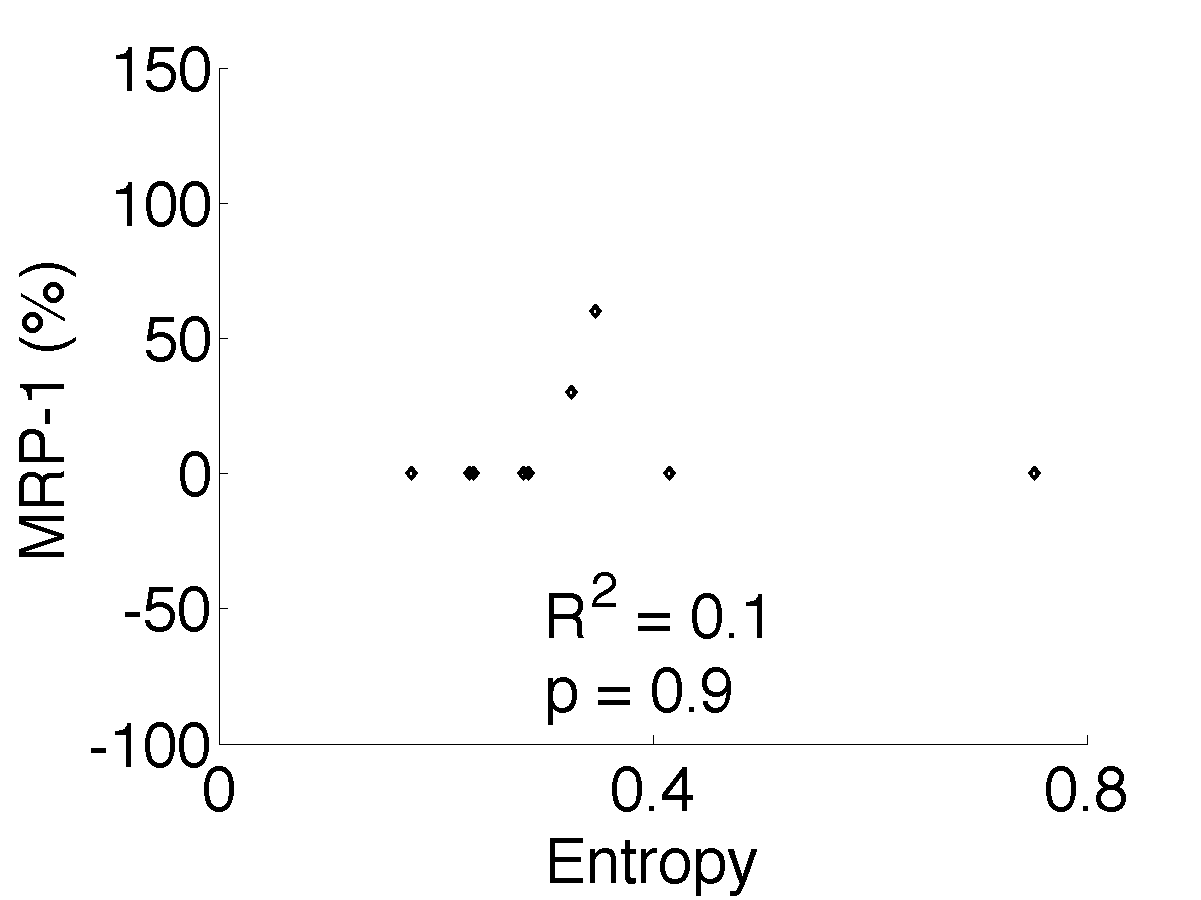


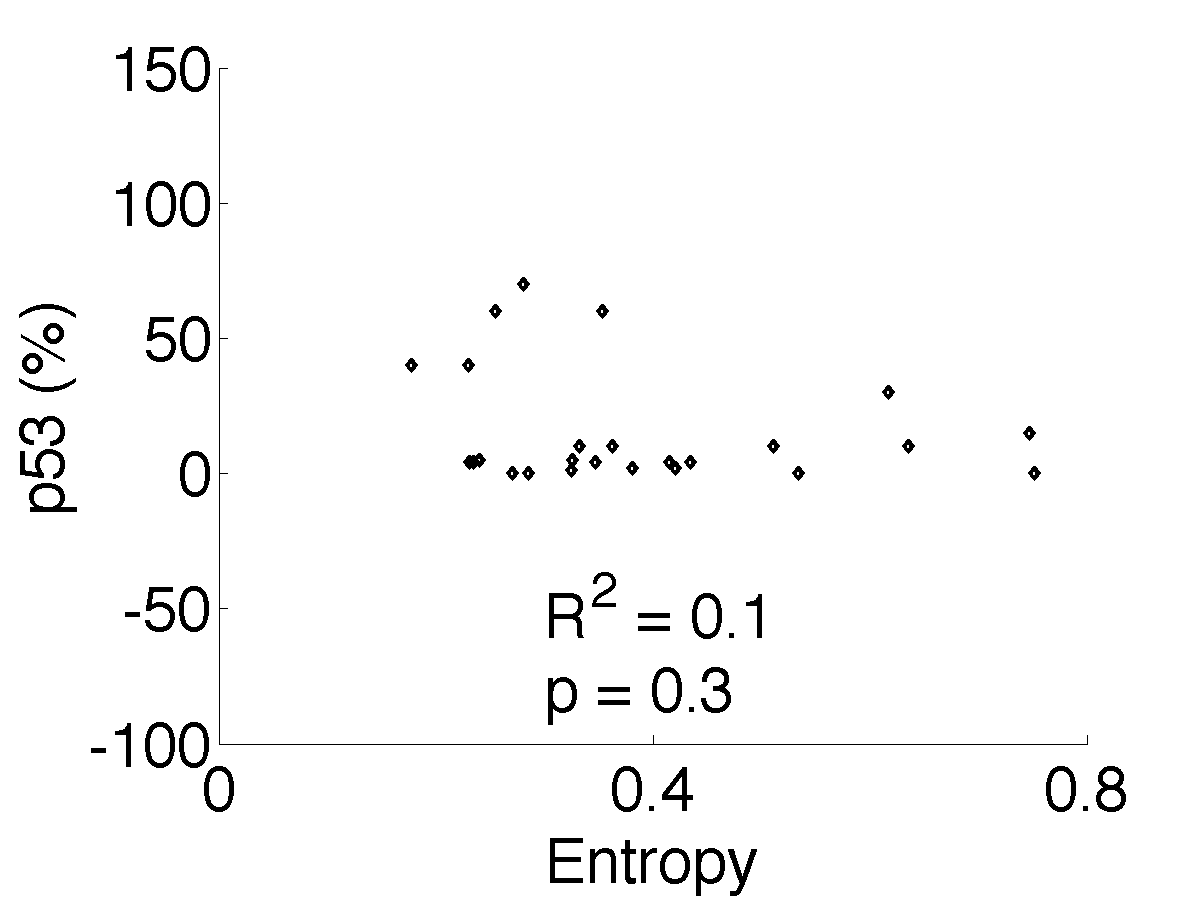


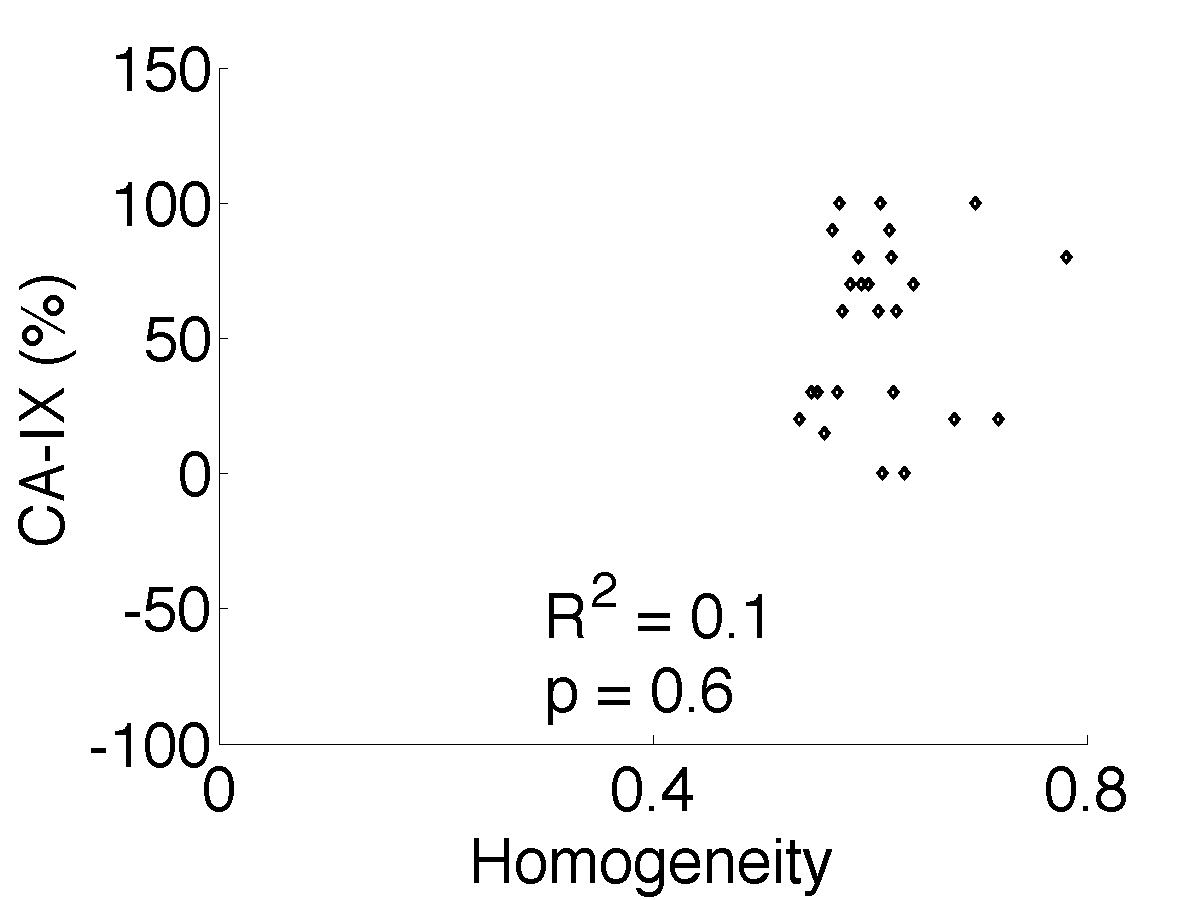


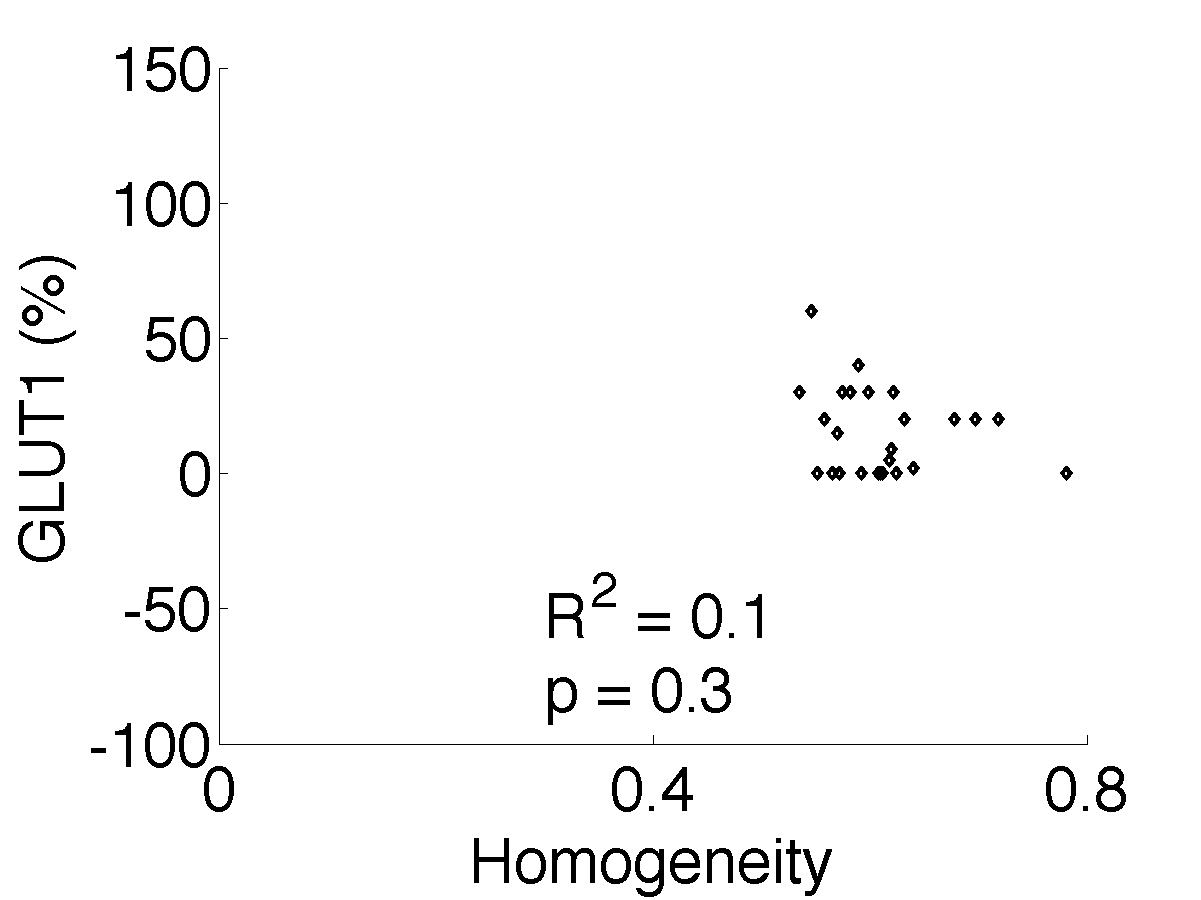


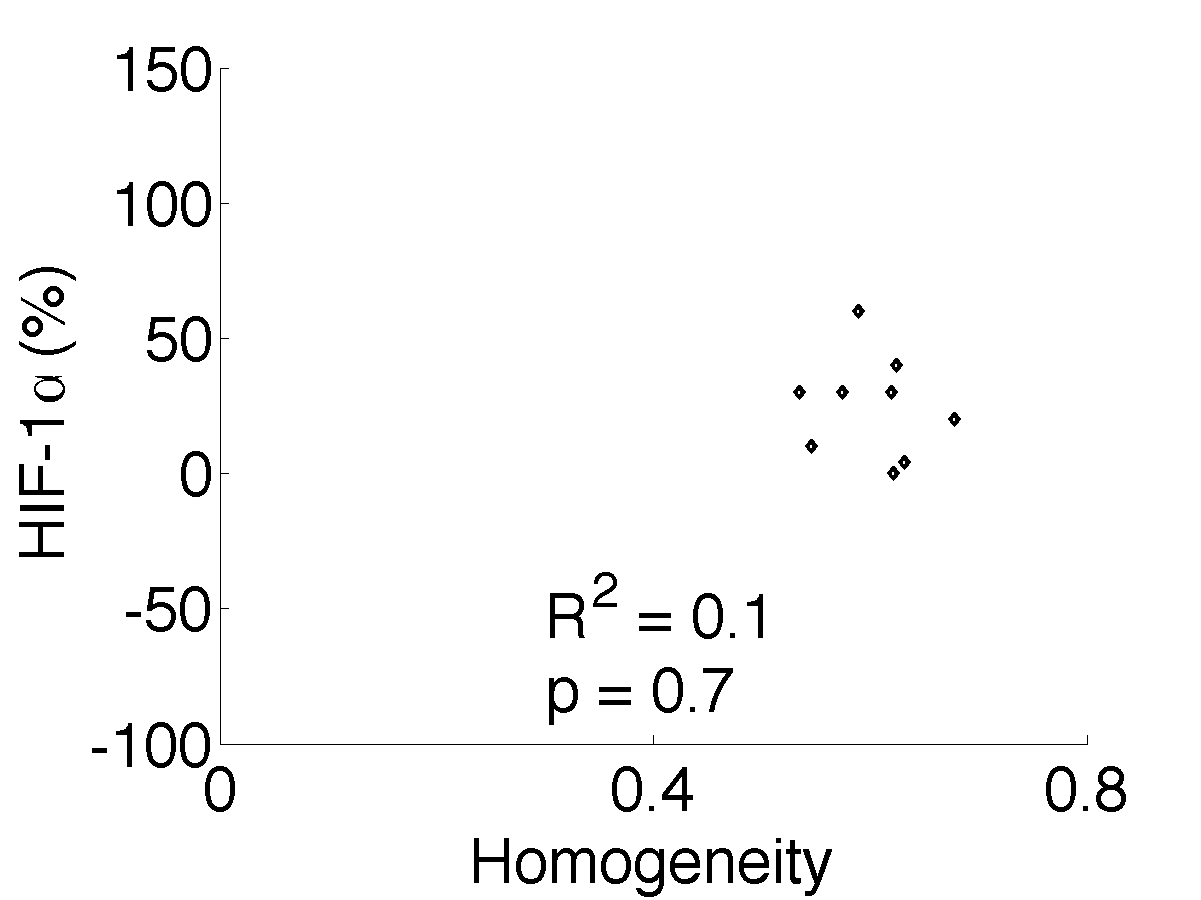


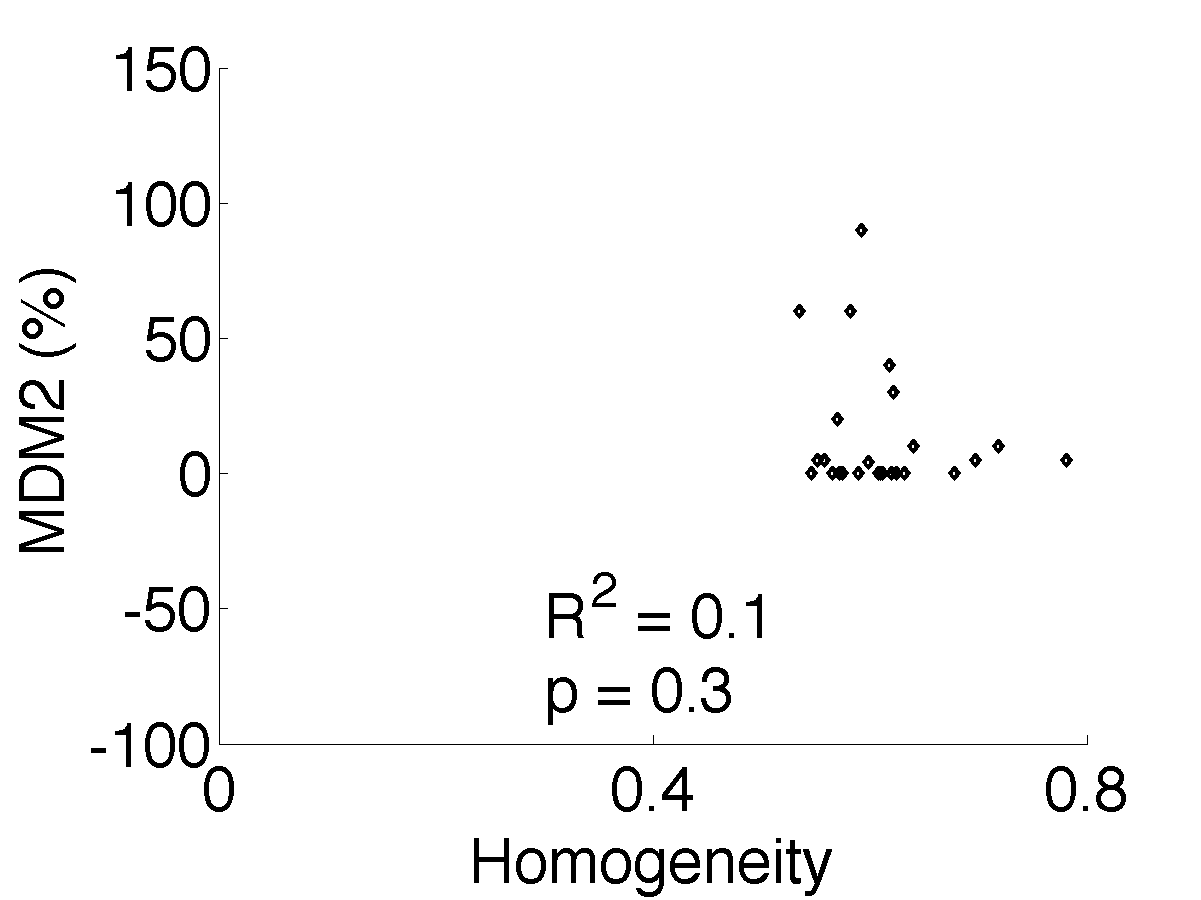


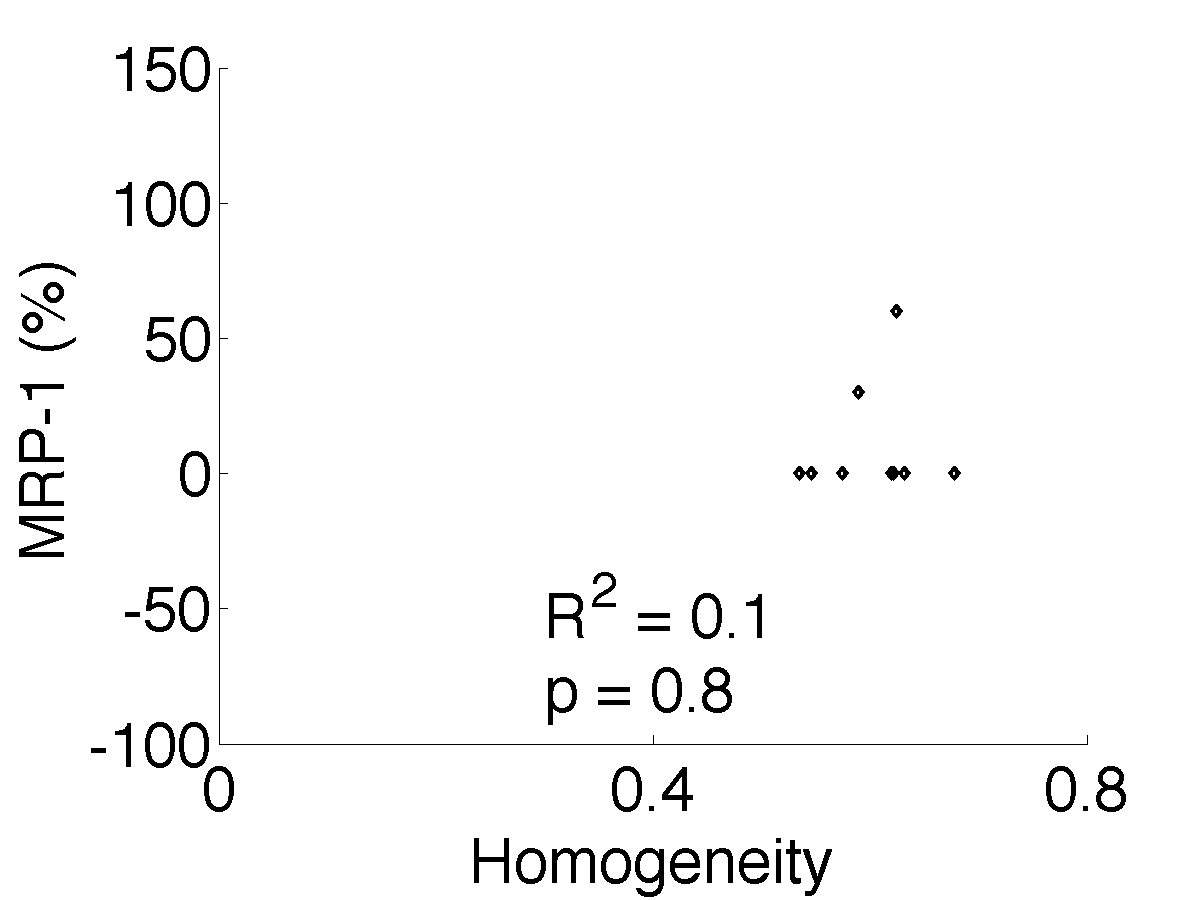


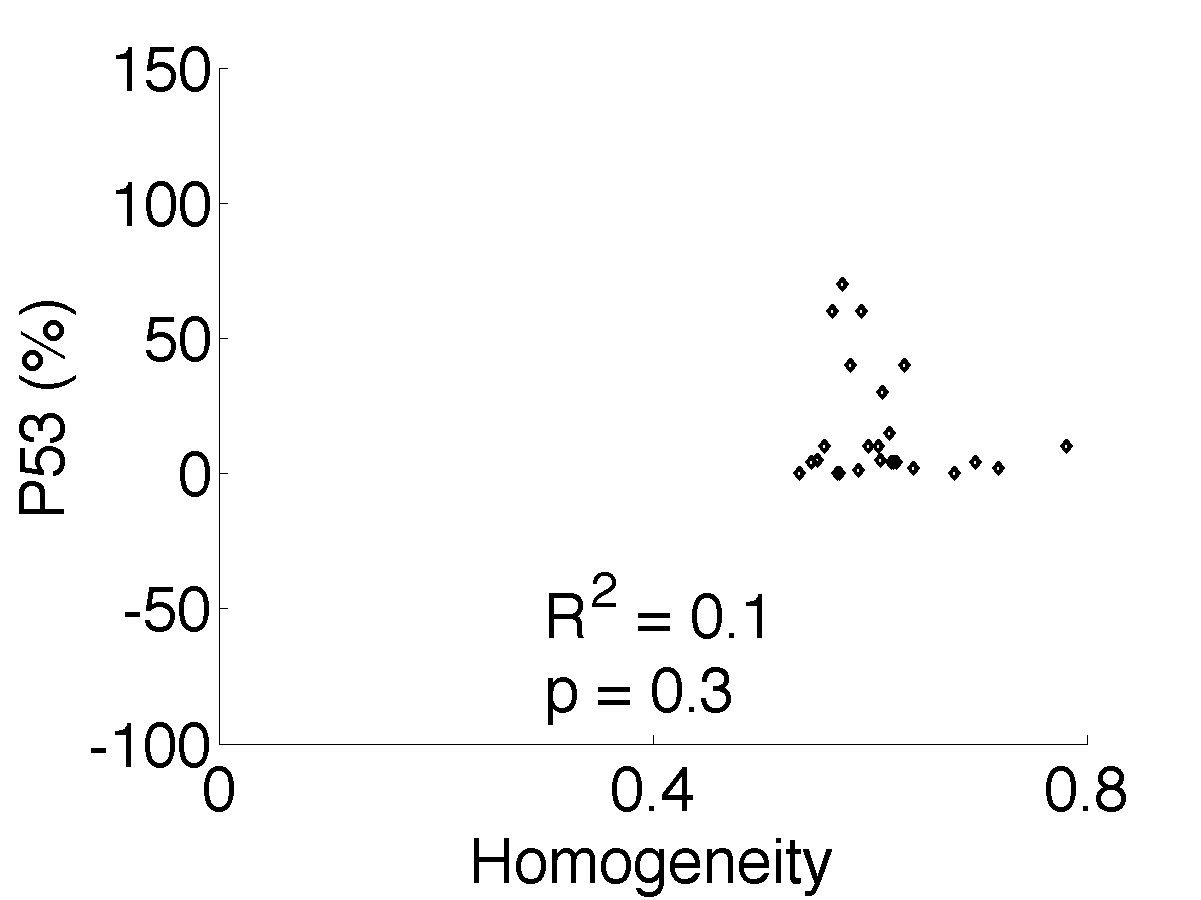

Supplement: S1 Fig — The 95% confidence interval is rendered. (DOC) [file pone.0132953.s001.doc]
