# Supplementary material for: Cholangiocarcinoma: Correlation between Molecular Profiling and Imaging Phenotypes
Source: PLoS One. 2015 Jul 24;10(7):e0132953. doi: 10.1371/journal.pone.0132953 (PMC4514866; doi:10.1371/journal.pone.0132953)
Supplement: S1 Table — (DOC) [file pone.0132953.s002.doc]

**Supplementary data**

**S1 Table.** Hypoxia related markers and immunohistochemical staining data.

| **Protein** | **Number of tumors with adequate staining** | **Positive staining** | **Description** |
| --- | --- | --- | --- |
| **VEGF^1^** | 24 (96%) | 16 (67%) | In tumor biology both the EGF(R) and the VEGF(R) pathway are constitutively activated due to genetic abnormalities and ongoing tumor-associated hypoxia. |
| **EGFR^1^** | 24 (96%) | 18 (75%) |
| **CA-IX^2^** | 25 (100%) | 23 (92%) | Carbonic anhydrase IX (CA-IX) is a transmembrane protein and is a tumor-associated carbonic anhydrase isoenzyme, which is overexpressed in solid tumors. |
| **HIF-1α^1^** | 9 (36%) | 7 (78%) | Hypoxia-inducible factor 1-alpha (HIF-1α): a pivotal mediator of cellular and systemic responses to hypoxia. |
| **P53^3^** | 25 (100%) | 7 (28%) | P53 is a tumor suppressor protein, which is hypoxia induced. |
| **MDM2^4^** | 25 (100%) | 5 (20%) | Mouse double minute 2 homolog (MDM2): a multifunctional oncoprotein that acts as a negative regulator of p53 tumor suppressor and plays a role in hypoxia-mediated VEGF upregulation. |
| **CD24^5^** | 9 (36%) | 5 (55%) | CD24 is a cell adhesion molecule and effector of HIF-1α. |
| **MRP-1^6^** | 9 (36%) | 2 (22%) | Multidrug resistance-associated protein 1 (MRP-1): involved with HIF-1α in multidrug resistance. |
| **GLUT1^2^** | 25 (100%) | 13 (52%) | Glucose transporter 1 (GLUT1): effector of HIF-1α. |

^1^ Marmé D, Fusenig N. Tumor angiogenesis : basic mechanisms and cancer therapy: Springer-Verlag; 2008.

^2^ Perez-Sayans M, Supuran CT, Pastorekova S, Suarez-Penaranda JM, Pilar GD, Barros-Angueira F, et al. The role of carbonic anhydrase IX in hypoxia control in OSCC. Journal of oral pathology & medicine : official publication of the International Association of Oral Pathologists and the American Academy of Oral Pathology. 2013;42(1):1-8.

^3^ Lee JH, Jin Y, He G, Zeng SX, Wang YV, Wahl GM, et al. Hypoxia activates tumor suppressor p53 by inducing ATR-Chk1 kinase cascade-mediated phosphorylation and consequent 14-3-3gamma inactivation of MDMX protein. J Biol Chem. 2012;287(25):20898-903.

^4^ Zhou S, Gu L, He J, Zhang H, Zhou M. MDM2 regulates vascular endothelial growth factor mRNA stabilization in hypoxia. Mol Cell Biol. 2011;31(24):4928-37.

^5^ Thomas S, Harding MA, Smith SC, Overdevest JB, Nitz MD, Frierson HF, et al. CD24 is an effector of HIF-1-driven primary tumor growth and metastasis. Cancer Res. 2012;72(21):5600-12.

^6^ Ding Z, Yang L, Xie X, Xie F, Pan F, Li J, et al. Expression and significance of hypoxia-inducible factor-1 alpha and MDR1/P-glycoprotein in human colon carcinoma tissue and cells. Journal of cancer research and clinical oncology. 2010;136(11):1697-707.
