# Supplementary material for: Cholangiocarcinoma: Correlation between Molecular Profiling and Imaging Phenotypes
Source: PLoS One. 2015 Jul 24;10(7):e0132953. doi: 10.1371/journal.pone.0132953 (PMC4514866; doi:10.1371/journal.pone.0132953)
Supplement: S2 Table — (DOC) [file pone.0132953.s003.doc]

**Supplementary data**

**S2 Table**. Imaging features definitions.

| **Imaging feature name** | **Imaging feature Definition** |
| --- | --- |
| **Tumor - Liver Difference, Maximum** | The maximum of the arterial and portal venous phase tumor - liver difference scores |
| **Attenuation Heterogeneity, Maximum** | The maximum of the arterial and portal venous phase attenuation heterogeneity scores |
| **Internal Arteries** | The presence or absence of discrete arteries within the tumor |
| **Capsule** | A discrete rim of enhancement circumscribing the tumor on the portal venous phase imaging (may completely or partially circumscribe the tumor) |
| **Hypodense Halo** | A discrete rim of hypoattenuatioin circumscribing the tumor on both arterial and portal-venous phase imaging. A hypodense halo on arterial phase that became hyperdense on portal-venous phase was considered a capsule. |
| **Internal Septa** | The presence or absence of discrete septa with-in the tumor on the portal-venous phase of imaging |
| **Enhancement Pattern** | The enhancement pattern of the tumor (mosaic, target, homogenous) |
| **Tumor Margin Score, Maximum** | The maximum of the arterial and portal-venous phase tumor margin score |
| **Liver Capsule, Abutment** | Does the tumor contact the liver capsule? |
| **Liver Capsule, Bulge** | Does the tumor bulge the liver capsule? |
| **Capsule retraction** | Does the tumor retract the liver capsule? |
| **Biliary dilatation** | Any biliary dilation? |
| **primary features** | |
| **Tumor Margin Score** | The Tumor Margin score was a qualitative assessment of the transition zone from tumor tissue to liver tissue, scored from 0 to 4, where '0' is a perfectly demarcated tumor with a sharply defined transition between tumor and liver, and a score of '4' had an infiltrating morphology with a broad ill-defined transition along the entire periphery of the tumor. |
| **Wash-Out** | The relative decrease in attenuation of the tumor from arterial phase to portal-venous phase imaging, graded from 0 to 4. |
| **Tumor - Liver Difference** | The degree to which the tumor attenuation differed from that of adjacent liver, graded from 0 to 4, where '0' indicates that the tumor is isodense to adjacent liver and is perfectly homogeneous, and a score of '4' indicates the attentuation of the tumor differed greatly from the adjacent background liver |
| **Attenuation Heterogeneity** | A qualitative measure of the dispersion of attenuation values within the tumor. For example, a score of '0' meant the tumor was of uniform attenuation, whereas a score of '4' meant the tumor had areas of both very high and low attenuation. |
