# Supplementary material for: Cholangiocarcinoma: Correlation between Molecular Profiling and Imaging Phenotypes
Source: PLoS One. 2015 Jul 24;10(7):e0132953. doi: 10.1371/journal.pone.0132953 (PMC4514866; doi:10.1371/journal.pone.0132953)
Supplement: S3 Table — (DOC) [file pone.0132953.s004.doc]

**Supplementary data**

**S3 Table:** Relationship between texture features and protein expression levels by linear regression.

| **Hypoxia markers** | **Contrast** | | | **Correlation** | | | **Energy** | | | **Entropy** | | | **Homogeneity** | | |
| --- | --- | --- | --- | --- | --- | --- | --- | --- | --- | --- | --- | --- | --- | --- | --- |
|  | **β** | **P-value** | **R^2^** | **β** | **P-value** | **R^2^** | **β** | **P-value** | **R^2^** | **β** | **P-value** | **R^2^** | **β** | **P-value** | **R^2^** |
| **CA-IX** | -0.1 | 0.5 | 0.1 | -0.3 | 0.2 | 0.3 | 0.1 | 0.6 | 0.1 | -0.1 | 0.7 | 0.1 | 0.1 | 0.6 | 0.1 |
| **HIF-1α** | 0.1 | 0.8 | 0.1 | 0.1 | 0.9 | 0.1 | -0.2 | 0.6 | 0.1 | 0 | 1 | 0 | -0.2 | 0.7 | 0.1 |
| **P53** | 0.2 | 0.4 | 0.2 | -0.2 | 0.4 | 0.2 | -0.2 | 0.4 | 0.1 | -0.2 | 0.3 | 0.1 | -0.2 | 0.3 | 0.1 |
| **MDM2** | 0.2 | 0.4 | 0.2 | -0.1 | 0.6 | 0.1 | -0.2 | 0.4 | 0.1 | -0.1 | 0.6 | 0.1 | -0.2 | 0.3 | 0.1 |
| **MRP-1** | -0.1 | 0.7 | 0.1 | -0.1 | 0.7 | 0.1 | 0.1 | 0.9 | 0.1 | 0.1 | 0.9 | 0.1 | 0.1 | 0.8 | 0.1 |
| **GLUT1** | 0.3 | 0.2 | 0.3 | -0.1 | 0.6 | 0.1 | -0.2 | 0.3 | 0.1 | -0.2 | 0.4 | 0.1 | -0.2 | 0.3 | 0.1 |
